# A Facial Foundation Model for Clinical Biomarker Prediction and Real‐World Mobile Deployment

**DOI:** 10.1002/advs.77861

**Published:** 2026-10-01

**Authors:** Tingfeng Xu, Huixuan Xu, Li Lin, Fei Wang, Yaodong Ding, Shuangqiao Liao, Xingyu Chen, Jichen Wen, Hongqiang Zhao, Jing Hao, Weixiang Zhao, Gong Zhang, Shiguang Shan, Yong Zeng, Hu Han, Minxian Wang

**Affiliations:** ^1^ China National Center for Bioinformation Beijing China; ^2^ Beijing Institute of Genomics Chinese Academy of Sciences Beijing China; ^3^ University of Chinese Academy of Sciences Beijing China; ^4^ Institute of Computing Technology Chinese Academy of Sciences Beijing China; ^5^ Beijing Anzhen Hospital Capital Medical University Beijing China; ^6^ Cardiovascular Disease Initiative Broad Institute of MIT and Harvard Cambridge MA USA; ^7^ Department of Cardiology Beijing Daxing Hospital Beijing China; ^8^ Cardiovascular Research Center Massachusetts General Hospital Boston MA USA

**Keywords:** coronary artery disease, deep learning, face analysis, heart disease prediction, medical genetics, predictive biomarker, risk prediction models, software deployment

## Abstract

While most biomarkers currently rely on invasive laboratory testing, which limits large‐scale or repeated screening, scalable non‐invasive methods could transform population screening and personalized health management. Facial photographs, as a ubiquitous and non‐invasive data source, offer such potential but remain underexplored for clinical biomarker prediction. Existing supervised approaches are constrained by the availability of clinically labeled data, whereas general‐purpose vision models are optimized for non‐clinical tasks and may inadequately capture subtle, multiscale, and spatially distributed clinical facial features. To address these limitations, we developed MedicalFaceFound, a facial foundation model pretrained on over 10 million images and evaluated across 62 biomarkers spanning eight physiological systems. It performed best among the Swin‐Large and ResNet18 for 45 (73%) biomarkers and generalized across four independent external cohorts (median Pearson's *r* = 0.172), with strong performance for RBC, eGFR, and HDL‐C (median *r* = 0.478, 0.442, and 0.430, respectively). MedicalFaceFound also outperformed polygenic risk score models for 14 of 26 biomarkers, and face‐estimated cardiovascular biomarkers were strongly associated with coronary stenosis (AUC = 0.66). The model retained predictive performance with 400 labeled samples and was feasible to deploy as a smartphone application, supporting scalable, non‐invasive biomarker assessment and personalized risk screening.

## Graphical abstract

MedicalFaceFound is a facial foundation model developed through three‐stage progressive pre‐training, adapting representations from general vision to facial structure and clinical phenotypes. Using a single facial image, it estimates 62 biomarkers across multiple physiological systems and supports downstream risk prediction for cardiovascular, metabolic, lipid, and hematologic conditions, enabling scalable, non‐invasive health assessment.

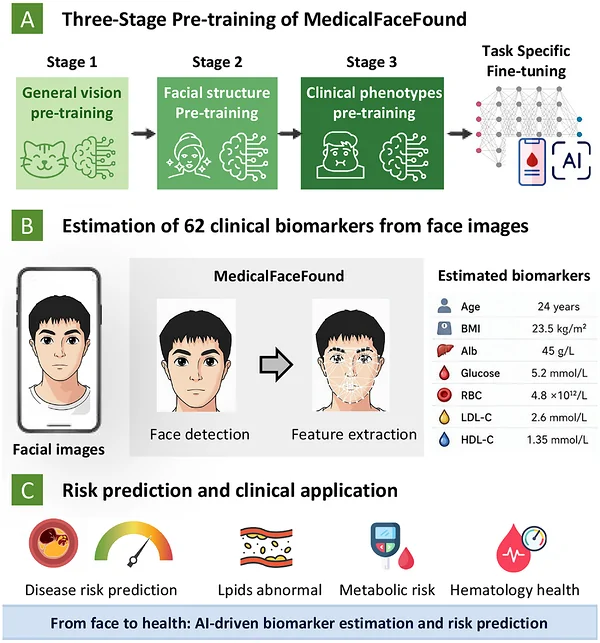

## 1 Introduction

Quantifying internal physiological states is fundamental to disease prevention, early diagnosis, and personalized health management [1]. Clinical biomarkers are essential indicators that span multiple organ systems and are widely utilized for diagnosis [2], risk stratification [3], and informed therapeutic decision‐making [4]. Yet, most biomarkers require invasive sampling and laboratory‐based testing, which limits accessibility for large‐scale screening and frequent monitoring, particularly in resource‐constrained settings. Approaches that enable non‐invasive, widely deployable biomarker assessment and disease risk evaluation could therefore transform population health screening and personalized care [5–8].

Facial photographs are ubiquitous, low‐cost, and non‐invasive images. Facial features have been recognized as reflecting health and disease status; for example, renal dysfunction may manifest as facial edema or pallor [9], and decompensated cirrhosis may lead to yellowish facial discoloration due to elevated serum bilirubin levels [10]. Advances in computer vision now enable the extraction of complex phenotypic features from facial images at scale [11–14]. Vision foundation models, trained on large and diverse unlabeled image datasets, offer a robust framework for learning transferable representations [12, 15] and have demonstrated success in medical image analysis across various diseases [16–19].

Across visual prediction tasks, supervised learning enables strong endpoint‐specific modeling when labeled data are available in sufficient quantity for model training, whereas unsupervised transfer learning [20,21] and self‐supervised learning [22] can improve domain adaptation and representation learning when target‐domain labels are limited. Clinical facial analysis has similarly been advanced primarily through task‐specific supervised and transfer‐learning approaches. Pioneering studies, including Deep Facial Diagnosis [23,24] and DeepGestalt [25,26], established the feasibility of using facial images for disease and genetic syndrome classification, while subsequent studies such as facial CAD detection [27] and FaceAge [28] extended facial phenotyping to cardiovascular diagnosis and cancer prognosis. Despite these important advances, existing models are generally optimized for narrow facial analysis tasks [29–32], or individual‐specific tasks or disease applications [26,27,33] which rely on endpoint‐specific supervision. This gap motivates the development of a progressively adapted, self‐supervised facial foundation model for multi‐system clinical phenotyping, which would leverage both advantages of the foundation model for general image and face feature representation and increase the performance for task‐specific applications.

Foundation models for broad, clinical facial phenotyping remain underdeveloped. Existing approaches face three major challenges. First, task‐specific supervised models must learn both facial representations and biomarker prediction functions from limited clinically labeled samples, which makes it difficult to reliably identify subtle facial features or heterogeneous health‐related signals [23,34]. Second, general‐purpose vision models are pretrained to learn broadly transferable object‐ and scene‐level semantics and therefore tend to prioritize globally salient patterns over fine‐grained local variations, with limited performance on task‐specific applications [29,32,35]. Consequently, subtle, multiscale, and spatially distributed facial cues associated with clinical phenotypes may be insufficiently represented. Third, general face‐domain models are commonly optimized for identity discrimination or non‐clinical facial attributes and are typically trained on in‐the‐wild images [29,36]. Such models are not specifically designed to capture variation in health‐related phenotypic features, resulting in both objective and data‐domain gaps when transferred to clinical facial phenotyping.

To address these limitations, we developed MedicalFaceFound, a facial foundation model that adopts a progressive pre‐training strategy to sequentially adapt representations from general vision to facial structure and ultimately to clinical facial phenotypes. Specifically, MedicalFaceFound was pretrained in three stages: ImageNet‐22K was used to learn general visual representations; VGGFace2‐HQ was used to adapt these representations to facial structure and localized facial patterns; and a clinical facial‐image dataset was used to further align the model with clinical acquisition characteristics and health‐related facial phenotypic variation (Figure 1a). Fine‐tuned on 62 clinical biomarkers, MedicalFaceFound was compared with conventional architectures (ResNet18 and Swin‐Large) using 4 independent cohorts in terms of prediction accuracy, label efficiency, stability with repeated images, and risk assessment for coronary stenosis (Figure 1b). The prediction performance was also compared to polygenic risk score (PRS) models in an external cohort with genetic data available (Figure 1c). To evaluate the feasibility of smartphone‐based image application through the MedicalFaceFound model, we implemented MedicalFaceFound through a smartphone application with server‐side inference and recruited an independent validation cohort in which facial photographs were acquired using a smartphone camera (Figure 1d). Collectively, this study establishes MedicalFaceFound as a scalable, non‐invasive framework for clinical biomarker prediction with broad implications for precision medicine and digital health.

**FIGURE 1:**
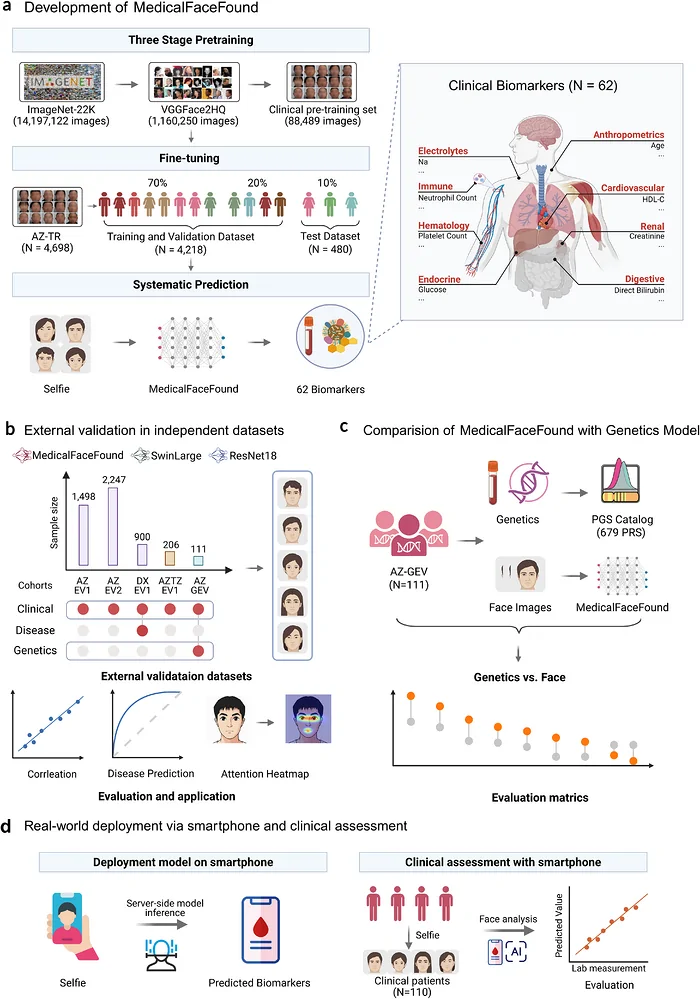
Study design. (a) Overview of model pre‐training and fine‐tuning. To develop the foundation model, we use a progressive general‐to‐clinical pre‐training strategy, integrating 15.4 million general images and clinical facial photographs. After pre‐training, the model was fine‐tuned and evaluated on the AZ‐TR dataset (N = 4,698) to predict systemic biomarkers from facial images, encompassing the anthropometric, cardiovascular, hematology, renal, endocrine, electrolyte, digestive, and immune systems. (b) External validation across five independent datasets. The fine‐tuned models for 62 biomarkers were externally validated in five independent cohorts: AZ‐EV1 (N = 1,498), AZ‐EV2 (N = 2,247), AZTZ‐EV (N = 206), DX‐EV (N = 900), and AZ‐GEV (N = 111). For each cohort, the models were applied to predict all systemic biomarkers, and performance was evaluated by Pearson's *r*. (c) Comparison of systemic biomarker prediction performance between MedicalFaceFound and polygenic risk score (PRS) models in an independent cohort AZ‐GEV based on Pearson's *r*. (d) Proof‐of‐concept evaluation of MedicalFaceFound using smartphone‐acquired facial photographs in the independent APP‐EV (N = 110) cohort. Images were acquired using a smartphone application and processed via server‐side inference. The facial icons were generated using ChatGPT's image‐generation capabilities (OpenAI), while additional icons were sourced from Flaticon. The overall figure was assembled and finalized using BioRender.com.

## 2 Results

### 2.1 MedicalFaceFound development

We developed a facial foundation model, MedicalFaceFound, using self‐supervised learning (SSL) with a progressive general‐to‐clinical strategy. We implemented the general‐to‐clinical pre‐training pipeline utilizing ImageNet‐22K (14,197,122 images) [37], VGGFace2‐HQ (1,160,250 images) [36,38], facial photographs from the Chinese Anzhen Training Cohort (88,489 images) (Figure 1a and Figures S1–S3). In the first stage, the Swin‐Large Transformer backbone was pretrained on ImageNet‐22K with 14.2 million images to capture general‐purpose visual features. In the second stage, the model was further adapted using approximately 1.16 million high‐resolution facial images from the VGGFace2‐HQ dataset. In the final stage, domain‐specific pre‐training was conducted using 88 489 clinical facial photographs from a Chinese hospital dataset to adaptively learn features for the focal population (Figure 1a, Figure S1a, and Methods). The progressive general‐to‐clinical pre‐training strategy achieved the lowest reconstruction loss among the evaluated strategies on clinical facial images (Figure S1b).

To fine‐tune MedicalFaceFound for predicting 62 biomarkers routinely tested in a healthcare system across eight physiological systems (Table S1), we used 4,698 individuals with linked hospital records from the AZ‐TR‐unfiltered dataset (median age 60.0, IQR, 54.0‐67.0, AZ‐TR), and each with more than three frontal facial photographs and corresponding biomarker measurements. Fine‐tuning for these biomarkers was performed with task‐specific adapter layers (Methods and Figure S1c).

### 2.2 MedicalFaceFound Predicts Clinical Biomarkers Across Internal and External Cohorts

We evaluated the performance of the MedicalFaceFound model for estimating 62 biomarkers covering eight clinically physiological systems and compared its performance with the baseline Swin‐Large and ResNet18 models, in an internal validation cohort subset (10%, N = 480) from the AZ‐TR cohort, and four external validation cohorts (AZ‐EV1, N = 1,498; AZ‐EV2, N = 2,247; AZTZ‐EV, N = 206; DX‐EV, N = 900) (details in Methods, Tables S2 and S3).

We first examined the predictability of clinical biomarkers across systems based on MedicalFaceFound. Among the eight physiological systems, anthropometric biomarkers showed the highest predictive accuracy in both the internal cohort (median Pearson's *r* = 0.698, IQR = 0.600–0.817) and external cohorts (median Pearson's *r* = 0.702, IQR = 0.641–0.794), followed by renal biomarkers (internal cohort: median Pearson's *r* = 0.277, IQR = 0.192–0.392; external cohorts: median Pearson's *r* = 0.315, IQR = 0.192–0.429). In contrast, electrolyte biomarkers showed the weakest predictive performance in both the internal cohort (median Pearson's *r* = 0.141, IQR = 0.108–0.185) and external cohorts (median Pearson's *r* = 0.030, IQR = 0.018–0.068) (Figure 2a and Table S4).

**FIGURE 2:**
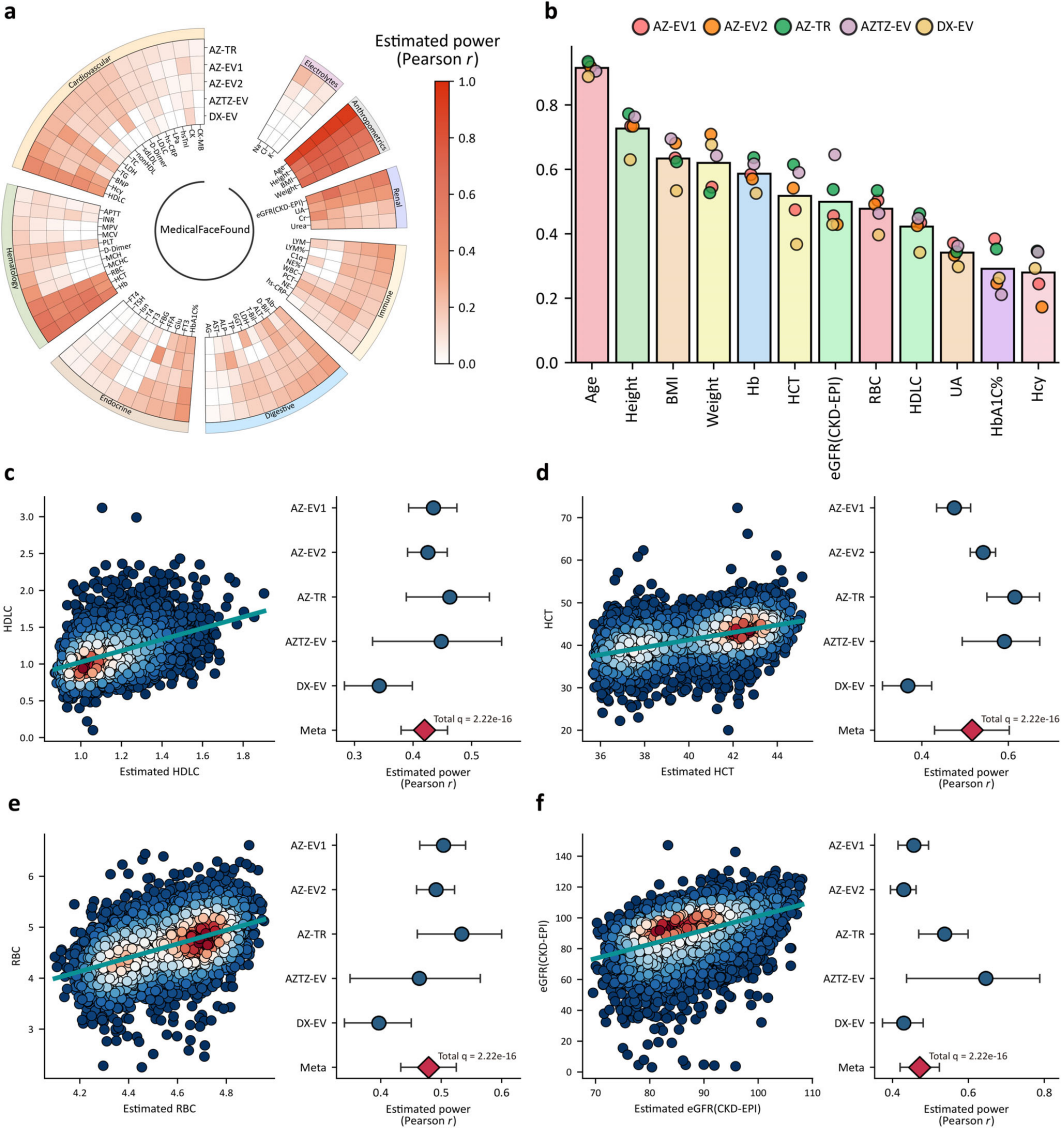
MedicalFaceFound estimates multi‐system biomarker values across independent cohorts. (a) MedicalFaceFound performance across multiple centers for each physiological system, with color intensity indicating Pearson's *r*. (b) Performance of the most performed estimation biomarkers (with Pearson's *r* > 0.3 in the internal set and in at least one external validation cohort) in both internal and external validation cohorts from our model. (c‐f) Estimation performance for HDL‐C (c), HCT (d), RBC (e), and eGFR(CKD‐EPI) (f) across internal and external cohorts. In each sub‐figure, the left panels show measured values plotted against MedicalFaceFound‐derived values, with the fitted trend line indicating the direction of association. Right panels show cohort‐specific Pearson's *r* estimates with 95% CIs. Points indicate Pearson's *r*, and horizontal error bars indicate 95% CI; red diamonds indicate the pooled meta‐estimate across cohorts. Reported q values denote the significance of the pooled association after multiple‐testing correction. Abbreviations: BMI, body mass index; Hb, haemoglobin; HCT, haematocrit; eGFR, estimated glomerular filtration rate; CKD‐EPI, Chronic Kidney Disease Epidemiology Collaboration; RBC, red blood cell count; HDL‐C, high‐density lipoprotein cholesterol; UA, uric acid; HbA1C, glycated haemoglobin; Hcy, homocysteine; CI, confidence interval.

Among the 62 clinical biomarkers, 15 achieved Pearson's *r* > 0.3 in the internal test cohort with comparable performance in the external cohorts (median Pearson's *r* = 0.441, IQR: 0.291–0.627). Among them, 12 biomarkers reached Pearson's *r* > 0.30 in at least one external cohort, distributing in Anthropometrics (n = 4; age, height, weight, and BMI), Hematology (n = 3; HCT, Hb, and RBC), Cardiovascular (n = 2; HDL‐C and Hcy), Renal (n = 2; UA and eGFR), and Endocrine (n = 1; HbA1C), respectively (Figure 2b). These findings indicate that MedicalFaceFound can extract facial‐image features that are statistically associated with measured biomarkers across several physiological systems.

Among these, we found HDL‐C (internal cohort: Pearson's *r* = 0.462, 95% CI 0.388–0.530; external cohort: median Pearson's *r* = 0.430, IQR 0.404–0.438), HCT (internal cohort: Pearson's *r* = 0.615, 95% CI 0.550–0.672; external cohort: median Pearson's *r* = 0.508, IQR 0.448–0.554), RBC (internal cohort: Pearson's *r* = 0.534, 95% CI 0.460–0.600; external cohort: median Pearson's *r* = 0.478, IQR 0.447–0.494), and eGFR(CKD‐EPI) (internal cohort: Pearson's *r* = 0.537, 95% CI 0.469–0.599; external cohort: median Pearson's *r* = 0.442, IQR 0.429–0.503) emerged as the most robust biomarkers estimated by MedicalFaceFound, showing strong agreement and consistent meta‐analytic Pearson's *r* estimates between estimated and measured values across both internal and external cohorts (Figure 2c–f). The results for the remaining biomarkers are shown in Figure S4. Complementary performance metrics, including MAE, MSE, MAPE, NRMSE, and R^2^, were also calculated and are reported in Table S5. These results indicate that facial photographs contain predictive information associated with lipid, hematologic, and renal biomarker values.

Next, we compared the predictive performance of MedicalFaceFound with the two widely used baselines, Swin‐Large [35] and ResNet18 [39]. We found that MedicalFaceFound outperformed Swin‐Large and ResNet18 in both internal and external cohorts. In the internal cohort, MedicalFaceFound (median Pearson's *r* = 0.197, IQR: 0.104–0.291) outperformed Swin‐Large (median Pearson's *r* = 0.168, IQR: 0.071–0.243) and ResNet18 (median Pearson's *r* = 0.108, IQR: 0.041–0.210) for the prediction of 62 biomarkers. Further, in the four external cohorts, MedicalFaceFound consistently outperformed (median Pearson's *r* = 0.172, IQR: 0.081–0.266) over Swin‐Large (median Pearson's *r* = 0.151, IQR: 0.057–0.246) and ResNet18 (median Pearson's *r* = 0.099, IQR: 0.033–0.182) (Figure S5a). Totally MedicalFaceFound outperformed the baselines for 45 out of the 62 biomarkers in the internal cohort (Figure S5b and Table S6). Collectively, these findings show that MedicalFaceFound extracts associated information from facial images for a broad spectrum of clinical biomarkers, with comparatively stronger performance for several cardiovascular and renal biomarkers.

MedicalFaceFound‐derived biomarker estimation contained information beyond readily measured demographic and anthropometric variables (age, sex, height, weight, and BMI), we used multivariable models to decompose the variance explained by the included features. For 26 of the 58 biomarkers, MedicalFaceFound‐estimated values remained significantly associated with the corresponding laboratory measurements after adjustment for these measured variables. Within the fitted models for these biomarkers, the MedicalFaceFound component accounted for a median of 65.8% (IQR, 51.3–75.5%) of the total variance explained by all of the included features. These results indicate that MedicalFaceFound provided associated information beyond the measured demographic and anthropometric variables (Figure S6 and Table S7).

We next performed three complementary analyses to assess whether the MedicalFaceFound‐derived biomarker signals extended beyond information attributable to age, sex, and related demographic and anthropometric factors. For each biomarker, we first assessed the incremental variance explained by the MedicalFaceFound‐estimated value beyond a baseline model including age, sex, height, weight, and BMI. Across the internal test cohort and two external validation cohorts, 43 of 58 biomarkers (74.1%) showed a significant incremental R^2^ in at least one cohort after joint FDR correction (Figure S7 and Table S8). In a complementary analysis, model‐derived and laboratory‐measured values were separately residualized for the baseline model variables, after which 40 biomarkers (69.0%) retained a significant residual correlation in at least one cohort. Although the adjustment attenuates the significance for some biomarkers, the two different adjustments generally yield the same result (Figures S8 and S9). Among the 44 biomarkers significant in the unadjustment analysis, 20 were significant in both the adjustment and unadjustment analyses in all three cohorts and 15 in at least one cohort (Figure S10). Representative findings spanned multiple physiological systems and included Hb, HCT, RBC, HDL‐C, HbA1C, and eGFR (CKD‐EPI), with generally concordant directions across datasets despite variation in statistical significance (Figure S10 and Table S8). We then evaluated eight representative biomarkers within subgroups defined by age, sex, BMI category, and coronary stenosis status. While some biomarkers exhibited variation across strata, the model also maintained significant performance across all strata (Figure S11 and Table S9). Collectively, these findings suggest that, for a substantial subset of biomarkers, the model can capture additional clinical signals beyond the demographic variables.

### 2.3 MedicalFaceFound Predictive Performance Compared to Polygenic Risk Scores

To compare MedicalFaceFound prediction with genetics‐based prediction for biomarker level estimation, we curated 679 published multi‐ancestry polygenic risk scores (PRSs) for the above 26 clinical biomarkers from the PGS Catalog [40]. These PRSs were originally developed in independent studies for different target populations and analytical purposes, spanning ancestry‐specific, multi‐ancestry, and cross‐ancestry prediction settings. We then computed individual‐level PRSs in the AZ‐GEV cohort, in which the genetic data were available (N = 111; median age, 59.0 years; IQR, 52.0–65.5; Table S3). For each biomarker, the best‐performing PRS, evaluated by Pearson's correlation coefficient (*r*), was selected to represent genetics‐based information and was compared with MedicalFaceFound‐derived prediction. Across the 26 biomarkers, MedicalFaceFound outperformed the best‐performing PRS for 14 traits, including BMI, HDL‐C, haemoglobin and eGFR(CKD‐EPI), which are clinically relevant indicators of cardiometabolic, haematological and renal health (Figure 3a,b, Figure S12 and Table S10). Moreover, to quantify the relative contribution of genetic and facial information for the prediction, we fitted multivariable models for each of the 26 biomarkers, including both the best‐performing PRS and the MedicalFaceFound score as predictors. Facial features accounted for a larger independent proportion of explained variance than PRSs for 14 biomarkers, whereas PRSs showed a larger contribution for 12 biomarkers (Figure 3c and Table S11). These results indicate that for 14 (53.8%) of the 26 evaluated biomarkers, MedicalFaceFound has better performance than that captured by the selected polygenic risk scores, supporting facial imaging as an accessible modality for biomarker estimation.

**FIGURE 3:**
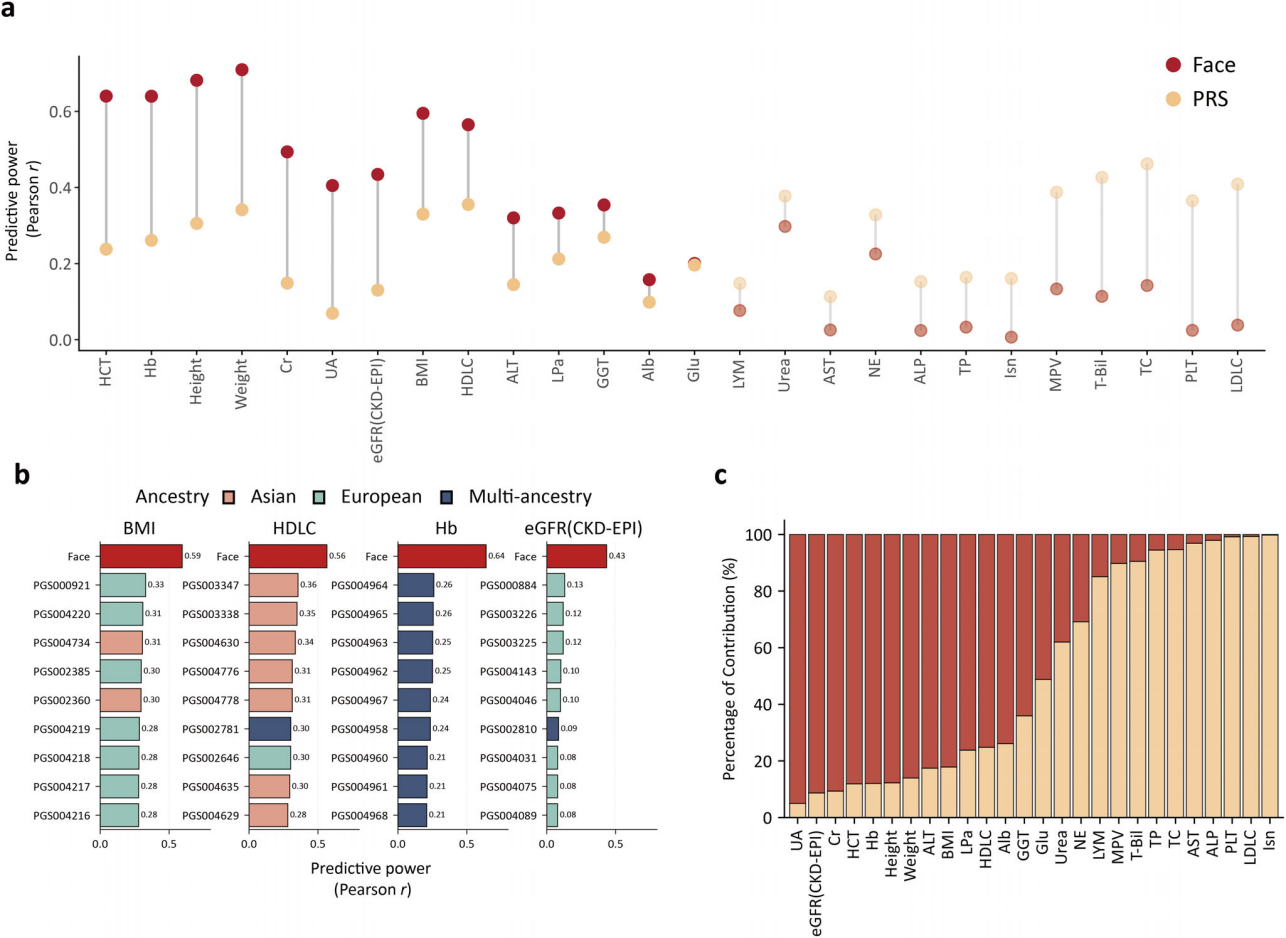
Comparison between MedicalFaceFound and polygenic risk scores for biomarker prediction. (a) Predictive performance of facial representations and the multi‐ancestry polygenic risk scores (PRSs) across 26 biomarkers in the external AZ‐GEV cohort (N = 111). Performance is reported as Pearson's *r* between estimated and measured values. Facial representations were defined as the estimated MedicalFaceFound values for each biomarker. PRS for each biomarker was selected from a broad range of available PRS published in the PGS Catalog, and showed the performance with the best predictive power in the AZ‐GEV cohort. Red points denote predictions from facial features, whereas orange points denote PRS‐based predictions; grey lines connect the two estimates for each phenotype. (b) Benchmarking of face‐based prediction against individual PRSs for four representative traits: body mass index (BMI), high‐density lipoprotein cholesterol (HDL‐C), haemoglobin (Hb), and estimated glomerular filtration rate calculated using the CKD‐EPI equation. Horizontal bars show Pearson's *r* for Face and for the best top nine PRSs from the PGS Catalog. Bar colors denote the ancestry group in which each PRS was developed, including Asian, European, and multi‐ancestry cohorts. For each biomarker, only the nine PRSs with the highest predictive performance are shown. (c) Relative contribution of facial representations and PRSs to phenotype prediction across biomarkers. Stacked bars show the percentage of variance explained by each information source, with facial features shown in red and PRSs shown in orange.

### 2.4 MedicalFaceFound‐Estimated Cardiovascular Biomarkers Preserve Stenosis‐Associated Information and Improve Risk Stratification

Next, we assessed the clinical values of MedicalFaceFound‐estimated biomarker values compared with laboratory measurements by examining their associations with coronary stenosis severity (≥50% vs. <50%), a clinical diagnosis indicator for coronary artery disease, in AZ‐TR and DX‐EV cohorts with available coronary angiography (CAG) data (Figure 4a). Across 16 cardiovascular‐related biomarkers, model‐estimated values were consistent with laboratory measurements (Figure S13 and Table S12), with particularly similar patterns for six key biomarkers (HDL‐C, LDL‐C, TG, TC, HbA1C, hs‐TnI) (Figure 4b). For example, for HDL‐C, a biomarker of established clinical relevance, facial estimation (OR/SD 0.705, 95% CI: 0.579–0.858, P < 0.001) showed associations consistent with those obtained from laboratory measurements (OR/SD 0.856, 95% CI: 0.749–0.978, P = 0.022), both having higher levels in the control group. In contrast, for LDL‐C, since most participants in the studied cohorts were cardiology inpatients receiving statin‐based lipid‐lowering therapy in AZ‐TR and DX‐EV cohort, leading to a negative association for laboratory‐measured LDL‐C (OR/SD 0.726, 95% CI: 0.636‐0.829, P<0.001), whereas face‐estimated LDL‐C preserved the expected positive association (OR/SD 1.445, 95% CI: 1.252‐1.668, P<0.001). The discordant associations suggest that facial model‐estimated LDL‐C may reflect a composite phenotype related to coronary stenosis rather than contemporaneous circulating LDL‐C levels alone.

**FIGURE 4:**
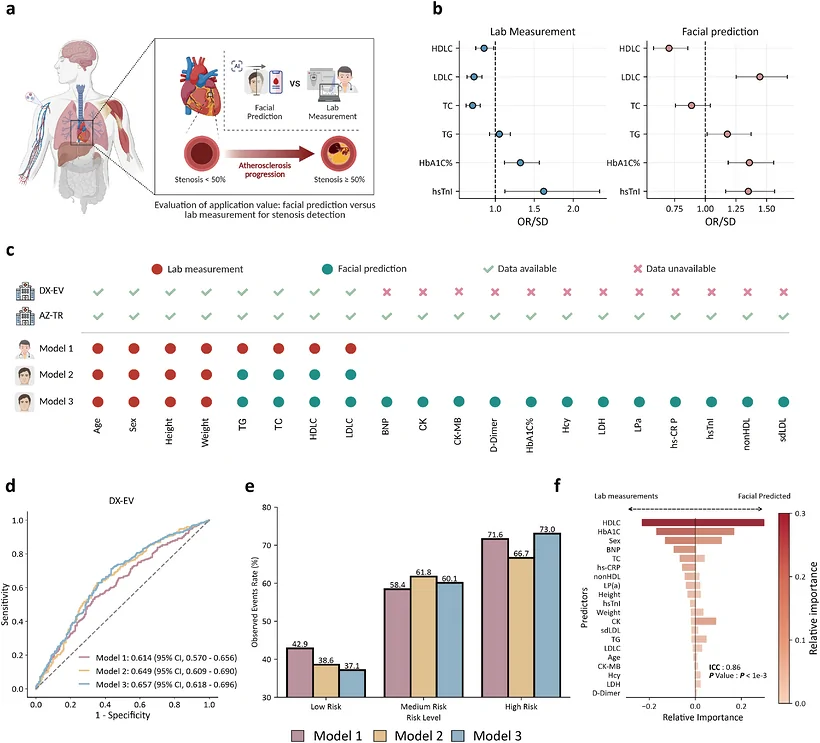
Evaluation of MedicalFaceFound estimated biomarkers for coronary stenosis detection. (a) Conceptual framework illustrating the comparison of MedicalFaceFound estimated biomarkers with laboratory‐measured biomarkers for detecting coronary stenosis (≥50%). (b) Associations of six cardiovascular biomarkers (HDL‐C, LDL‐C, TG, TC, HbA1C, hs‐TnI) with coronary stenosis, presented as odds ratios per SD (OR/SD) with 95% CI from both facial predictions and laboratory measurements with a logistic regression adjusted for age, sex, height, weight, and BMI. (c) Data availability and feature composition across models and cohorts. Rows indicate cohort or model configurations (Models 1–3), and columns represent included variables. Features of the model are represented as laboratory measurements (red circles) or facial predictions (green circles). Model 1 was developed with 4 basic laboratory‐measured lipid profiles (LDL‐C, HDL‐C, TC, TG). Model 2 was replaced with 4 MedicalFaceFound‐estimated biomarker values. Model 3 was developed with 16 face‐derived cardiovascular biomarkers. (d) The area under the curve (AUC) for the three models in the DX‐EV cohorts. (e) Coronary stenosis prevalence across high‐ (>80%), medium‐ (20%‐80%), and low‐score (<20%) strata defined by each model in the DX‐EV cohort. (f) Relative importance of selected MedicalFaceFound‐estimated biomarker values compared with their laboratory‐measured counterparts, with intraclass correlation (ICC) indicating concordance between modalities. For Age, Sex, Height, and Weight, laboratory‐measured values were used. Relative importance was calculated as the variance explained by each focal predictor compared with the total variance explained by all predictors in the model. Figure 4a was created using BioRender.com.

Next, to evaluate the risk stratification potential of MedicalFaceFound‐estimated biomarker values, we developed and compared three coronary stenosis risk prediction models using different combinations of MedicalFaceFound‐estimated and laboratory‐measured biomarker values. The lab‐measured basic model (Model 1) incorporated demographic and anthropometric variables (age, sex, height, weight) with 4 basic laboratory‐measured lipid profiles (LDL‐C, HDL‐C, TC, TG). The face‐estimated basic model (Model 2) replaced 4 basic laboratory‐measured lipids with face‐estimated counterparts. The face‐estimated advanced model (Model 3) expanded the MedicalFaceFound‐estimated factors to 16 face‐estimated cardiovascular biomarkers (Figure 4c). The models were trained in AZ‐TR, and we evaluated classification performance using the area under the curve (AUC) in the external DX‐EV cohort (Table S13). In the external DX‐EV cohort, face‐estimated basic model (Model 2) and face‐estimated advanced model (Model 3) displayed comparable and higher performance (AUC: 0.649, 95% CI: 0.609–0.690 vs. 0.657, 95% CI: 0.618–0.696) compared with lab‐measured basic model (Model 1) (0.614, 95% CI: 0.570–0.656) (Figure 4d). In the low‐score group (the bottom 20% of risk score), stenosis prevalence was 42.9%, 38.6%, and 37.1% for the lab‐measured basic model, face‐derived basic model, and face‐derived advanced model, respectively; for the high‐score group (the top 20% of risk score), the rates were 71.6%, 66.7%, and 73.0%, respectively. These results indicate that the estimated biomarkers from MedicalFaceFound can achieve risk stratification values comparable to those from laboratory measurements (Figure 4e).

Finally, feature importance analysis comparing MedicalFaceFound‐estimated biomarker values with lab‐measured biomarker values in AZ‐TR showed that the contributions of MedicalFaceFound‐estimated biomarker values were consistent with those of their laboratory‐measured counterparts, with an intraclass correlation coefficient (ICC) of 0.86 (95% CI, 0.68‐0.94) (Figure 4f and Table S14). Among them, HDL‐C, HbA1C, and TC were the most influential predictors in both modalities, underscoring their robust association with the risk of stenosis. Together, these results show that MedicalFaceFound‐estimated biomarker values were associated with coronary stenosis and had risk‐stratification performance comparable to the laboratory measured values in these cohorts.

### 2.5 MedicalFaceFound Delivers Reproducible Predictions and Superior Label Efficiency with Limited Training Data

A critical requirement for clinical deployment is model stability, ensuring that predictions remain consistent across multiple replicates. To evaluate this, we performed a test‐retest analysis from internal and external cohorts, using facial photographs repeatedly acquired from the same participant with minor variations in capture angle. MedicalFaceFound exhibited excellent stability, achieving higher intraclass correlation coefficients (ICCs) than Swin‐Large and ResNet18 across all cohorts. In the internal AZ‐TR cohort, MedicalFaceFound achieved an ICC of 0.879 (IQR: 0.838–0.931); in the external datasets, MedicalFaceFound achieved higher ICC compared with other models, with 0.914 (IQR: 0.883–0.947) in AZ‐EV1, 0.913 (IQR: 0.881–0.947) in AZ‐EV2, and 0.971 (IQR: 0.958–0.980) in DX‐EV (Figure 5a and Table S15), respectively. Across physiological systems, MedicalFaceFound maintained stable performance, with median ICCs of 0.911 (IQR: 0.903–0.951) in both internal and external cohorts, with low variance (Figure 5b; Table S16). For example, HDL‐C showed high stability in AZ‐TR (ICC of 0.905, 95% CI: 0.898–0.912), AZ‐EV1 (ICC of 0.945, 95% CI: 0.944–0.946), AZ‐EV2 (ICC of 0.946, 95% CI: 0.944–0.947), and DX‐EV (ICC of 0.977, 95% CI: 0.976–0.978), as shown in the Bland–Altman plots (Figure 5c–f and Table S15). These findings confirm that MedicalFaceFound yields stable, reproducible biomarker predictions supporting its estimates.

**FIGURE 5:**
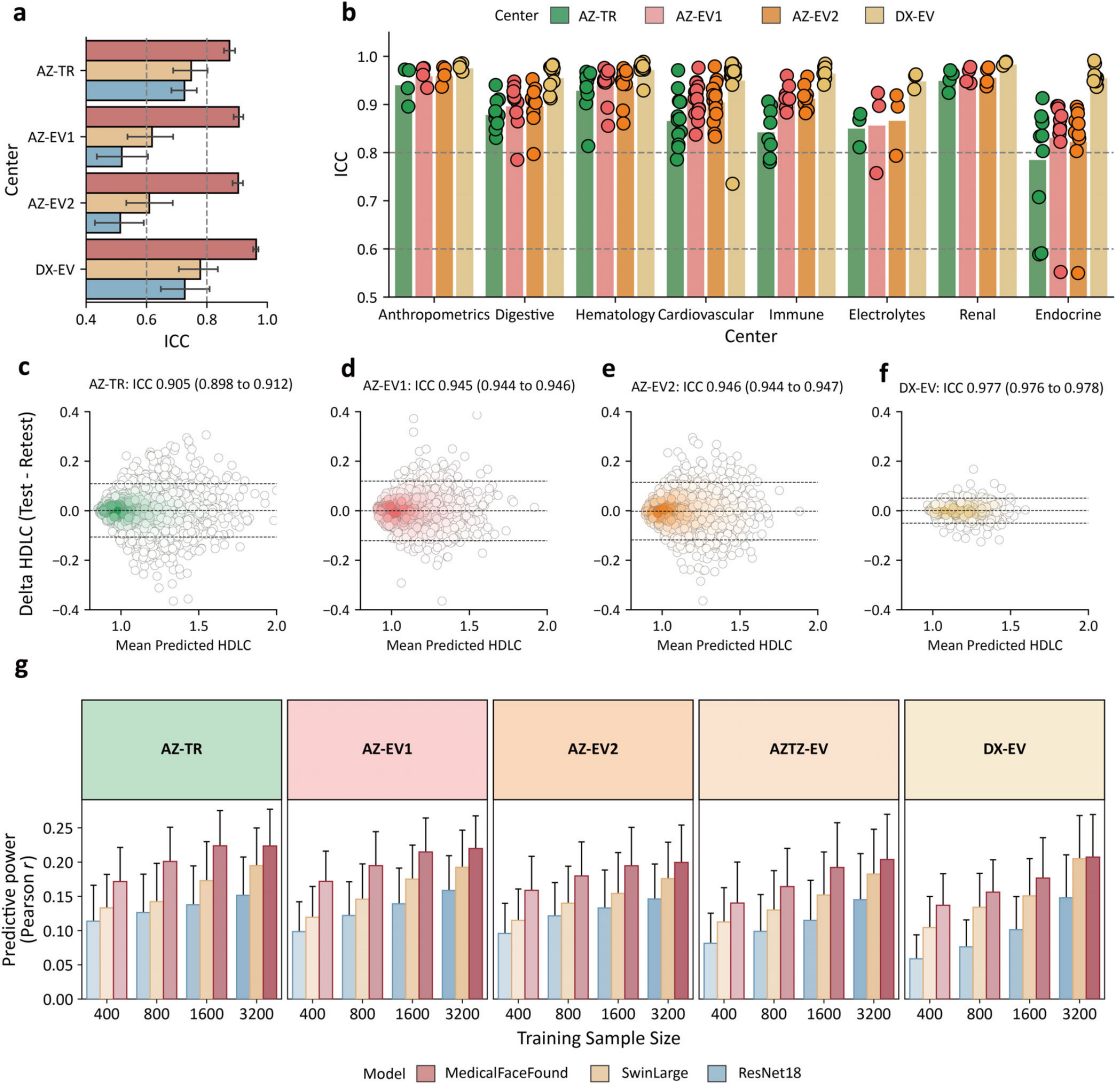
Reproducibility and generalizability of MedicalFaceFound estimated biomarkers across internal and external cohorts. (a) Intraclass correlation coefficients (ICCs) of three models on internal and three external cohorts (AZ‐TR, AZ‐EV1, AZ‐EV2, DX‐EV), showing test–retest reliability. Three models represent MedicalFaceFound and two baselines, Swin‐Large and ResNet18 models. MedicalFaceFound, Swin‐Large, and ResNet18 are indicated by pink, beige, and light blue boxes, respectively. The same color coding applies to both panels a and g. (b) MedicalFaceFound performance across eight physiological systems on internal (AZ‐TR) and three external (AZ‐EV1, AZ‐EV2, DX‐EV) cohorts, evaluated by ICC. (c–f) Bland–Altman plots of HDL‐C predictions in four cohorts, with dashed lines indicating the mean of the differences with 95% limits of agreement (mean ±1.96 SD); ICC values are shown for each cohort with 95% CIs. (g) Prediction accuracy (*r*) of 62 blood biomarkers under varying training data sample sizes (400 – 3200) for three models across cohorts, demonstrating the label efficiency of MedicalFaceFound with limited data.

Label efficiency refers to the amount of training data and labels required to achieve a target performance level for a given downstream task, which indicates the annotation workload for medical experts. We investigated the label efficiency of MedicalFaceFound by systematically evaluating training sample sizes from 400 to 3200, using 62 clinical biomarkers. Across all sample sizes settings, MedicalFaceFound consistently demonstrated superior label efficiency compared with Swin‐Large and ResNet18 across the internal and external cohorts (Figure 5g and Table S17). Notably, with as few as 400 samples, MedicalFaceFound (median Pearson's *r* = 0.125, IQR: 0.067–0.214) achieved substantially higher predictive performance than Swin‐Large (median Pearson's *r* = 0.085, IQR: 0.031–0.167) and ResNet18 (median Pearson's *r* = 0.055, IQR: 0.023–0.121) in the AZ‐TR cohort. To quantify data efficiency, we further estimated the proportion of training data required by MedicalFaceFound to match the performance of Swin‐Large or ResNet18. For eGFR (CKD‐EPI) and LYM, MedicalFaceFound reached equivalent accuracy using only 10% of labeled data. Superior efficiency was also observed for HbA1C (20%), BMI (29%), HDL‐C (55%), and Hb (63%) (Figure S14), respectively. These results demonstrate that MedicalFaceFound not only achieves stable and reproducible clinical predictions but also confers strong label efficiency, a property critical for developing scalable prediction in new domains or for novel biomarkers where annotated data are scarce.

### 2.6 MedicalFaceFound Learns Physiologically Plausible Facial Representations for Clinical Biomarker Prediction

To gain insights into how MedicalFaceFound achieved superior performance and label efficiency in clinical biomarker prediction, we conducted qualitative analyses of the pretext task used for self‐supervised pre‐training and of MedicalFaceFound's biomarker and stage‐specific decisions.

To evaluate the SSL pretext task, we curated internet‐sourced facial images covering both Asian and European populations, displaying diverse abnormalities, including peripheral facial paralysis, renal disease–associated edema, and Down syndrome, ensuring these facial images were not included in the pre‐training stage. We found that MedicalFaceFound was able to reconstruct pathological facial features with 75% of the image masked, across different types of facial deformities or abnormal traits, such as periorbital swelling, malocclusion, or macular lesions (Figure S15a). This demonstrates that MedicalFaceFound has learned to identify and infer the representation of health‐related areas by means of SSL, which contributes to performance and label efficiency in biomarker predictions. Beyond reconstruction‐based interpretation, we applied Grad‐CAM to the Swin‐Large backbone of MedicalFaceFound to visualize regions contributing to biomarker‐specific predictions (Figure S15b). For eGFR (CKD‐EPI), early stages captured global facial context, intermediate stages emphasized the eyes and cheeks, and the final stage focused on the periocular region. For Hb, later stages integrated signals from the entire face in addition to the eyes. For HDL‐C, salient contributions were largely concentrated around the periocular region. These findings suggest that MedicalFaceFound may rely on stage‐dependent facial regions that are potentially correlated with physiological characteristics when deriving diverse systemic biomarker estimations.

### 2.7 Real‐World Deployment of MedicalFaceFound via Smartphone for Clinical Use

To evaluate the feasibility of deploying the MedicalFaceFound model as a smartphone‐based application, we developed a WeChat‐based application through which standardized frontal facial photographs could be collected and submitted for server‐side inference. MedicalFaceFound generated estimates for 62 clinical biomarkers, and the resulting report was returned to the phone application side for display (Figure 6b).

**FIGURE 6:**
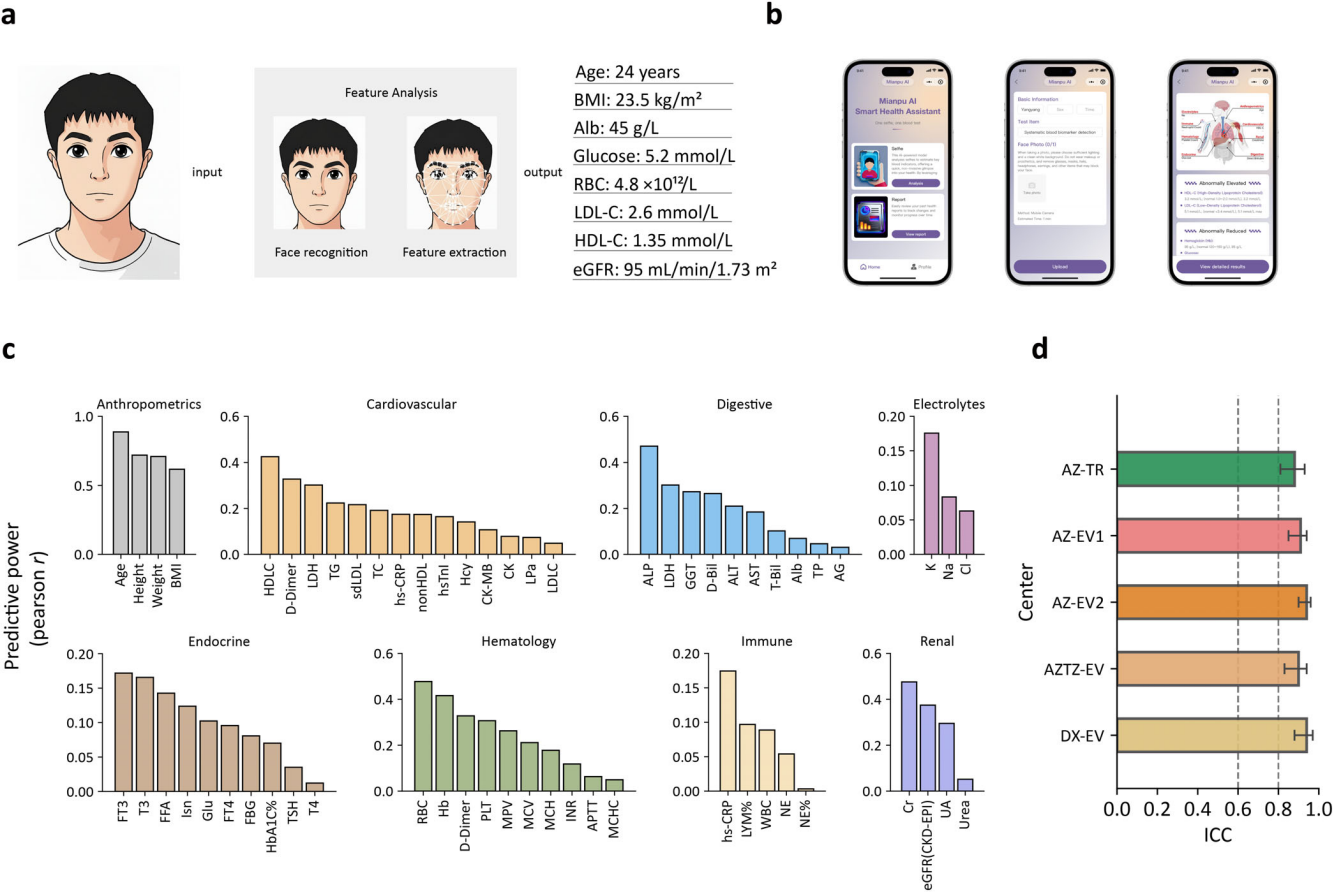
Smartphone deployment and validation of MedicalFaceFound. (a) Schematic of the MedicalFaceFound algorithm, which takes a single facial photograph as input. The model first localizes the face, then extracts quantitative features to predict 62 biomarkers. (b) Mobile application interface showing, from left to right, the home screen, photo upload/capture screen, and visualization of the interactive report. (c) Prediction accuracy (*r*) of MedicalFaceFound in the APP‐EV cohort (N = 110) using single photographs captured by smartphones. (d) Intraclass correlation coefficients (ICCs) comparing predictions from smartphone photographs with those from internal and four external validation cohorts across 57 biomarkers with available clinical records, demonstrating comparable accuracy between smartphone‐ and camera‐based photographs.

We conducted a pilot study in the cardiology department of Beijing Anzhen Hospital (APP‐EV), where frontal images were taken by a physician who was blinded to the study design, using the front‐facing camera of a mobile phone to take facial images. This cohort has 110 individuals with a median age of 60.0 (IQR, 55.0–67.0), and 26.4% are female (Methods and Table S18). For each individual, the medical records of 57 biomarkers were collected. Overall, after a systematic evaluation, the performance observed in APP‐EV was broadly consistent with that observed in the other validation cohorts (Figure 6c and Table S19), with consistency assessed by ICCs across AZ‐TR and the external cohorts AZ‐EV1, AZ‐EV2, AZTZ‐EV, and DX‐EV, with a median ICC of 0.910 (IQR, 0.900‐0.940) (Figure 6d and Table S20). These findings provide preliminary evidence that MedicalFaceFound can generate biomarker estimates from smartphone‐acquired facial photographs under physician‐assisted acquisition conditions. This deployment highlights the potential of MedicalFaceFound for rapid, low‐cost, and scalable integration into pre‐screening workflows and digital health monitoring platforms.

## 3 Discussion

In this study, we developed MedicalFaceFound, a facial foundation model based on a progressive general‐to‐clinical pre‐training strategy, and evaluated its ability to estimate clinical biomarker values across eight physiological systems using facial photographs from five hospital‐based cohorts in China. Our findings show that MedicalFaceFound can derive facial‐image‐based clinical signals that are statistically associated with measured biomarker values. The model‐derived estimates also provided clinical information complementary to the evaluated polygenic scores and were associated with coronary stenosis in the study cohort. Together, these results support facial imaging as a scalable input modality for non‐invasive biomarker estimation and risk stratification, without implying causal or mechanistic relationships between facial appearance and systemic physiology.

A central finding of our study is that MedicalFaceFound, using facial photographs, can estimate the values of a wide spectrum of routinely measured clinical biomarkers from facial photographs, with especially notable performance for biomarkers related to cardiovascular, renal, hematologic, endocrine, digestive, and immune systems. Previous studies of facial images have focused mainly on non‐medical domains such as person identity recognition and emotion analysis [29–32], with only limited work addressing health‐related outcomes, including aging [28], syndromic facial dysmorphisms [26], and coronary artery disease [27,33]. Our results help explain why facial images can be informative for clinical outcomes. The human face appears to encode measurable signals related to multiple internal physiological processes, rather than only disease‐specific visual patterns. In particular, the prediction of cardiovascular and metabolic biomarkers provides a biomarker‐level basis for prior facial image based coronary artery disease risk stratification, while the prediction of biomarkers across renal, endocrine, hematologic, digestive, and immune systems suggests that facial appearance may also reflect broader systemic aging and chronic physiological burden. These findings substantially extend previous disease‐specific observations by showing that facial images can support clinical biomarker prediction across multiple physiological systems. Notably, our work provides an explainable foundation for disease risk prediction based on facial images [27,]. The ability to predict biomarkers across different organ systems also allows the foundation model developed in this study to be applied to predict other chronic diseases, beyond cardiovascular disease, as shown in this study.

To examine whether these associations primarily reflected readily observable demographic and anthropometric characteristics, we performed complementary covariate‐adjusted analyses. For many biomarkers, model‐derived values provided incremental variance explained beyond age, sex, height, weight, and BMI, and the residual correlations remained statistically significant after adjustment for these demographic variables. These findings suggest that part of the facial‐model‐derived signal is not fully explained by these demographic factors examined.

More broadly, the present study was designed to evaluate clinical risk associations rather than causal relationships. The causal directionality of the observed relationships between facial features, clinical biomarkers, and disease‐related phenotypes cannot be established from the current data, and these relationships may be bidirectional or driven by shared determinants, including age, sex, BMI, genetic background, lifestyle, environmental exposures, comorbidities, aging, and treatment history and so on. Accordingly, our findings should not be interpreted as demonstrating that facial characteristics cause changes in systemic physiology, or that alterations in specific biomarkers directly cause the facial patterns identified by the model. Rather, the clinical value of these associations lies in their potential to support non‐invasive risk stratification and the earlier identification of individuals who may benefit from confirmatory laboratory testing or more comprehensive clinical care. This interpretation is consistent with the broader clinical application modeling framework, in which a feature may provide clinically useful information without being causally related to the associated outcome [41,42].

Although both genetic and environmental factors shape biomarker variation, previous studies have largely emphasized genetic contributions [43] while neglecting the substantial role of acquired exposures. Facial morphology, as a complex phenotype influenced by genetics and environment [44], provides a unique lens to evaluate personal health status, yet its variance explained compared with genetics remains unclear. By analyzing 679 PRS from 26 phenotypes, with the optimal multi‐ancestry PRS representing genetic effects, we found that facial signals achieved stronger prediction than genetic scores for some biomarkers and provided additive information when combined with genetic predictors. These results suggest that facial and genetic modalities capture overlapping but non‐identical components of biomarker and disease risk features. Genetic risk scores quantify inherited susceptibility, whereas facial‐model‐derived scores may contain information associated with a mixture of inherited and acquired characteristics, including age, adiposity, lifestyle, environmental exposures, comorbidities, medication use, treatment history, etc. The present analyses cannot disentangle the causal contributions of these factors. Given that genetic screening is increasingly used for disease risk assessment, the non‐invasive and convenient nature of facial imaging makes it a promising complementary modality. Our findings therefore support the potential of integrating facial information with genetic risk to improve biomarker prediction and individualized disease risk assessment, providing a more comprehensive framework for precision medicine.

Our study further underscores the clinical value of MedicalFaceFound‐estimated biomarker values. As the central indicators of physiological processes, biomarkers reflect disease progression and therapeutic interventions [45]. We found that Model‐derived cardiovascular biomarker estimation preserved clinically meaningful differences between health and disease states in the evaluation of coronary stenosis. Furthermore, incorporating facial model‐derived cardiovascular biomarker estimation improved stenosis risk evaluation beyond traditional risk factors. These results suggest that face‐estimated cardiovascular biomarkers may serve as non‐invasive predictors of CAD‐related risk and could be incorporated into disease prediction models to improve clinical risk stratification. Interestingly, we observed that participants receiving lipid‐lowering therapy exhibited reduced measured LDL‐C levels, whereas face‐estimated LDL‐C values remained elevated among individuals with coronary stenosis. This observation suggests that facial model‐estimated LDL‐C may represent an LDL‐C‐related composite facial phenotype rather than a direct surrogate of contemporaneous circulating LDL‐C levels. This composite phenotype may be shaped by multiple correlated clinical and physiological factors, including disease status, age, medication use, treatment history, metabolic characteristics, and other determinants. More broadly, MedicalFaceFound‐estimated biomarker values may capture biomarker‐associated composite phenotypes that integrate information related to the target biomarker and its correlated clinical context, thereby providing complementary value for clinical risk stratification and disease assessment.

A key strength of our work lies in the development of MedicalFaceFound as a dedicated foundation model for clinical facial phenotyping. Existing general vision models and internet‐scale face models are not specifically optimized as foundation models for clinical use, where disease‐relevant signals are often subtle, multiscale, and affected by image resolution, lighting, pose, acquisition devices, and collection conditions. Direct transfer from generic visual representations is limited by domain shifts between general visual data, in‐the‐wild facial images, and clinical facial images. The latter is enriched for health‐related phenotypic variation, whereas training solely from clinically labeled data is constrained by sample size and annotation availability. MedicalFaceFound addresses these challenges through progressive pre‐training from general visual representations to facial structural representations and finally to clinical facial representations. In MAE‐based benchmark analyses and downstream biomarker prediction tasks, MedicalFaceFound achieved stronger performance than conventional architectures, indicating that domain‐adapted facial representation learning is critical for extracting clinically meaningful signals. This model contribution is important because it provides a technical foundation for future studies of facial image based biomarker prediction, disease risk modeling, and clinical facial phenotyping.

MedicalFaceFound also demonstrated strong robustness and label efficiency, two properties that are essential for real‐world clinical translation. The model achieved excellent test‐retest reliability across internal and external cohorts and substantially outperformed existing models in stability across repeated images and different capture conditions. This is critical for clinical deployment, where facial photographs may vary in pose, angle, lighting, expression, device type, and image quality. Moreover, MedicalFaceFound maintained predictive performance even when trained with limited sample sizes, including settings with as few as 400 training samples. Such label efficiency suggests that foundation model pre‐training can reduce the dependence on extensive labeled datasets for each new biomarker or disease endpoint. This feature may be especially valuable for rare diseases, emerging infectious diseases, underrepresented populations, or other clinical scenarios where labeled facial and biomarker data are difficult to obtain. Grad‐CAM‐based interpretability analyses provided preliminary evidence that MedicalFaceFound attended to facial regions and patterns that may be biomarker‐specific, rather than relying exclusively on diffuse or nonspecific image features. However, Grad‐CAM visualizations identify image regions contributing to model estimations but do not establish causal or biologically mechanistic relationships between these regions and clinical biomarkers. These attribution patterns may also be influenced by model‐specific characteristics or imaging artifacts and should therefore be interpreted with care and the possible caveat.

Beyond the model development specific analyses, we conducted a preliminary evaluation using smartphone‐acquired facial photographs. The application supported facial image acquisition and report display, while model inference was performed on a remote server. The APP‐EV results provide proof‐of‐concept preliminary results for smartphone‐based image acquisition under physician‐assisted conditions but do not establish on‐device inference or comprehensive real‐world mobile deployability. This finding should not be interpreted as validation of unsupervised home‐based or population‐scale deployment. Future studies will expand smartphone‐based validation to larger and more diverse populations, multiple devices, and less controlled acquisition conditions, with particular attention to self‐capture feasibility and performance in large population screening settings.

Despite these advances, several limitations warrant consideration. The present observational and largely cross‐sectional study was designed to evaluate clinical associations rather than causal relationships. The directionality of the observed associations cannot be determined and may reflect bidirectional relationships or other determinants; therefore, facial‐model‐derived outputs should be interpreted as correlates rather than causal or proximal indicators of systemic pathophysiology. Data heterogeneity arising from differences in sampled populations, data‐acquisition devices, and collection protocols may impair model transferability. Expanding data collection to more diverse clinical populations or conducting center‐specific fine‐tuning will be important for improving model generalizability. The current study was limited to Chinese populations, and validation in other ancestral and geographic populations is needed to assess broader applicability. The smartphone‐based evaluation was conducted in a small (N = 110), single‐center cohort, with physician‐assisted image acquisition under relatively standardized conditions, which may limit generalizability to self‐capture settings. Larger multicenter studies involving diverse participants, smartphone devices, and acquisition conditions, especially those used in the smartphone selfie model, are therefore required in the future. Some external validation cohorts, particularly AZTZ‐EV (N = 206), also had relatively small sample sizes, which may limit statistical power and the precision of biomarker‐specific performance estimates. Larger and more diverse external cohorts are needed to further confirm the robustness of the findings. Grad‐CAM visualizations identify image regions contributing to model estimation but do not establish causal or biologically mechanistic relationships and may be even influenced by model‐specific characteristics or imaging artifacts. In addition, subtle and multiscale health‐related facial signals may be sensitive to image resolution, capture angle, illumination, and other acquisition conditions, which could affect prediction stability and generalizability in real‐world settings. Nevertheless, these limitations represent important directions for future research, and the present findings provide proof‐of‐concept evidence for the potential clinical value of facial‐image‐based biomarker prediction.

Collectively, our study establishes a new paradigm for precision health. A single facial photograph can serve as a non‐invasive, multidimensional signal associated with clinical physiology. By demonstrating broad biomarker predictability, complementarity with genetic risk, robust foundation‐model performance, and smartphone‐based deployment, MedicalFaceFound provides both biological evidence and technical infrastructure for future facial images based health assessment. These findings suggest that facial foundation models may support scalable biomarker estimation, chronic disease risk prediction, and population‐wide digital screening. With further validation and integration with clinical workflows with and appropriate ethical safeguards, facial‐image‐based biomarker prediction could become a practical component of personalized preventive healthcare.

## 4 Methods

### 4.1 Facial Image Acquisition

To develop MedicalFaceFound, we curated a large‐scale dataset comprising both public and clinical facial image datasets (Table S2). The public datasets included ImageNet‐22K [37] and VGGFace2‐HQ [36,38]. The ImageNet‐22K dataset, containing 14 197 122 real‐world images, was preprocessed according to standard protocols [37]to ensure a consistent image format and input scale while preserving the broad visual diversity of natural images. The VGGFace2‐HQ dataset (1 160 250 public facial images) was aligned and normalized according to standardized procedures [36,38] to generate consistently localized and scale‐normalized facial regions suitable for face‐domain pretraining.

The clinical facial datasets for pre‐training were collected from patients at Beijing Anzhen Hospital between 2021 and 2023 (AZ‐TR‐unfiltered, 129 769 images from all angles). To construct the dataset for Stage 3 pre‐training, we included only images that were either not linked to any clinical information due to failed simple ID mapping or assigned to the training set for subsequent fine‐tuning, resulting in a clinical pre‐training dataset of 88,489 images from all angles (Figure S3). All facial photographs were obtained by a physician who was blinded to the study design. A digital camera with a resolution of at least 20 megapixels was used in P mode, with an ISO sensitivity of 1600 and burst mode enabled. Images were captured in a quiet examination room with a plain white background and direct lighting to ensure privacy and consistency. Participants were instructed to keep their eyes open, maintain a neutral facial expression, and avoid accessories or hair covering facial features. Each participant was photographed in frontal, left and right 60° lateral, and downward‐facing positions (exposing the forehead), with 3–5 photographs per angle. These multiple angles were collected during pre‐training to capture facial information comprehensively. In the fine‐tuning stage for predicting 62 biomarkers, we used only a single frontal photograph, which achieved comparable predictive performance and offered greater practicality and translational value for downstream clinical applications.

To fine‐tune MedicalFaceFound to predict 62 biomarkers routinely tested in a healthcare system across eight physiological systems, we collected data from 4,698 individuals with linked hospital records from the AZ‐TR‐unfiltered dataset (N = 4698; median age 60.0 years; IQR 54.0–67.0; 26.4% female). The data was randomly divided into training, validation, and test sets in a 70:20:10 ratio.

For external validation, we additionally collected data from five independent cohorts using the same protocols, in which image acquisition was performed by different physicians blinded to the study content, and only frontal facial photographs were obtained. In the second half of 2023, we enrolled 1,498 participants at Anzhen Hospital (AZ‐EV1; median age 61.0 years, IQR 54.0–67.0; 24.4% female, 13,645 facial images). In 2024, an additional 2,247 participants were recruited at the same hospital (AZ‐EV2; median age 60.0 years, IQR 53.0–66.0; 22.2% female, 22,524 facial images). Between 2021 and 2022, 900 participants were recruited at Beijing Daxing Hospital (DX‐EV; median age 62.0 years, IQR 56.0–69.0; 49.9% female, 2,768 facial images). In 2025, we enrolled 206 participants from the Tongzhou branch of Anzhen Hospital (AZTZ‐EV; median age 61.0 years, IQR 56.0–68.0; 39.8% female, 206 facial images), and the AZ‐GEV cohort comprised 111 participants (median age 59.0 years, IQR 52.0–65.5; 30.6% female, 111 facial images), with one facial image per individual.

### 4.2 Biomarker Variables

We collected 62 clinical biomarkers covering risk factors across multiple organ systems (Table S1). For the internal cohort, a total of 62 biomarkers were collected in the AZ‐TR cohort, with the available sample size varying across biomarkers as determined by treating clinicians and standard institutional procedures. For blood sampling, venous blood was obtained in the morning after an overnight fast. Samples were stored at room temperature, protected from light, and delivered to the hospital laboratory within 30 min for routine assays. For the external cohorts, identical procedures were followed. For AZ‐TR and DX‐EV, we also collected coronary angiography (CAG) data to quantify stenosis, which further aided the development of a disease risk evaluation model. For AZ‐GEV, we further collected blood samples and used an Infinium Asian Screening Array (Illumina) to calculate biomarker genetic scores. Baseline characteristics of all clinical cohorts are summarized in Table S3.

Informed consent was obtained from all participants. The study was conducted in accordance with the principles of the Declaration of Helsinki and was approved by the Institutional Review Board of Anzhen Hospital (Ethical Approval No. 2022211X).

### 4.3 Facial Image Preprocessing

Facial photographs were first linked to the corresponding medical records by extracting patient identifiers through Optical Character Recognition (OCR) using “PaddleOCR” (v3.0.1) [46]. When OCR failed, manual correction was performed to ensure accurate matching. Once the patient identifier was extracted by OCR, it was used to associate all corresponding photographs with the same participant, after which the original uncropped image used for OCR was excluded from the final processed dataset. All photographs from the same participant were stored under a one‐way hashed identifier and linked to the corresponding de‐identified clinical data. Head pose was estimated using 6DRepNet [47], and only frontal images were retained for the prediction of 62 biomarkers, while images from all angles were used during pre‐training. The final dataset included only images successfully matched to hospital records based on participant name, patient ID, and timestamp. Blurry images were excluded. Remaining images were processed with DeepFace [48] for face detection, alignment, and cropping, and were subsequently standardized to 256 × 256 pixels for model training and evaluation (Figure S2). Only the cropped facial regions were retained in the processed dataset, thereby excluding any surrounding text or other potentially identifying non‐facial information.

### 4.4 MedicalFaceFound Architecture

MedicalFaceFound employs Swin‐Large as its encoder backbone [35], comprising four hierarchical stages with {2, 2, 18, 2} Swin Transformer blocks. Input facial photographs are partitioned into non‐overlapping 4 × 4 patches and projected into 192‐dimensional embeddings. Local window attention captures fine facial textures, while shifted‐window attention facilitates cross‐window and cross‐region information exchange, allowing the model to seamlessly integrate local details, regional appearance, and global facial morphology.

During pre‐training, the encoder backbone is paired with a lightweight reconstruction decoder (Figure S16). For downstream adaptation, the decoder is discarded, and the pre‐trained encoder is instead connected to task‐specific multilayer perceptron (MLP) heads for each biomarker prediction. The 62 biomarkers are estimated by 62 independently fine‐tuned models, each with a task‐specific head (Figure S17).

### 4.5 General‐to‐Clinical Self‐Supervised Pre‐Training

MedicalFaceFound was pre‐trained with a three‐stage General‐to‐Clinical curriculum to progressively adapt visual representations from generic natural images to facial morphology and then to clinical facial phenotypes (Figure S16). Each stage leveraged a primary dataset tailored to the intended representation level: Stage 1 utilized ImageNet‐22K to learn general visual priors; Stage 2 employed VGGFace2‐HQ to capture high‐resolution facial morphology and fine‐grained appearances; and Stage 3 used a subset of 88 489 facial images drawn from the train data set to adapt the model to standardized, patient‐domain clinical acquisition patterns (Figure 1a, Figure S1a, Figures S16, and S17).

To mitigate catastrophic forgetting during this progressive adaptation, a small proportion of data from preceding stages was retained as replay anchors. Specifically, Stage 2 incorporated a minor fraction of ImageNet‐22K images to preserve generic visual representations, while Stage 3 included sparse samples from both VGGFace2‐HQ and ImageNet‐22K to retain general visual and facial morphology priors. Thus, each curriculum step was primarily driven by the current‐stage dataset, with earlier‐stage samples serving solely as auxiliary anchors to stabilize representation transfer.

All three stages used Uniform Masking Masked Autoencoder (UM‐MAE) as the self‐supervised objective [49]. UM‐MAE masks image patches and trains the encoder‐decoder model to reconstruct the masked regions, encouraging representations that capture local texture, facial structure, and global context. A consistent training recipe was applied across stages, adjusted only for dataset‐specific input resolutions and window settings. Block‐wise masking with a high mask ratio increased the contextual reconstruction difficulty while reducing encoder computation. Furthermore, local target normalization mitigated the dominance of global color and illumination, rendering the reconstruction objective more sensitive to local facial structures.

Mild data augmentations, including random‐resized cropping, horizontal flipping, and weak color jitter, were applied to enhance robustness against variations in scale, framing, pose, and illumination without distorting clinically meaningful facial morphology. The model was optimized using AdamW [50] with a linear warm‐up and cosine learning rate decay. The warm‐up phase stabilized early updates following each domain transition, while cosine decay ensured stable convergence. Layer‐wise learning rate scaling was implemented to minimize drift in shallow, generic visual layers, allowing deeper layers to adapt to facial and clinical domains. From Stage 2 onward, L2‐SP [51] regularization was utilized to softly anchor the model to the previous‐stage checkpoint, further reducing representation drift during domain adaptation. Collectively, mixed‐data sampling and L2‐SP regularization provided complementary anti‐forgetting mechanisms at both the data and parameter levels. Complete details of experimental settings, including the software and hardware environments, training schedules, and stage‐specific hyperparameters, are provided in the Supplementary Information and Table S21.

### 4.6 Fine‐Tuning and Evaluation for Downstream Tasks

For downstream task adaptation, we used only the Swin‐L encoder from the foundation model as the backbone of MedicalFaceFound, discarding the UM‐MAE decoder. The backbone extracted high‐level facial representations, which were passed into a multilayer perceptron (MLP) to generate task‐specific predictions. For different downstream tasks, we used the MedicalFaceFound backbone and independently fine‐tuned biomarker‐specific task heads, resulting in 62 biomarker‐specific models, each optimized for a single biomarker task and producing outputs corresponding to the estimated quantitative values of the target biomarkers (Figure 1a and Figure S1c).

We conducted supervised fine‐tuning on all parameters of the Swin‐L backbone and the MLP for 50 epochs with a batch size of 32, using the AdamW optimizer. The first 10 epochs employed a linear warm‐up schedule, gradually increasing the learning rate from 0 to 5 × 10^−3^, followed by a cosine annealing decay schedule that reduced the learning rate from 5 × 10^−3^ to 1 × 10^−6^ over the remaining 40 epochs. We stopped the training early when the validation loss did not decrease for 16 consecutive epochs. After each epoch, models were evaluated on the validation set, and the checkpoint with the lowest validation loss was retained for subsequent testing on both internal and external cohorts.

### 4.7 Other Deep Learning Model Implementations

We implemented our proposed General‐to‐Clinical pre‐training strategy, leveraging the UM‐MAE self‐supervised pre‐training framework, to pretrain the Swin‐L backbone of MedicalFaceFound. We then compared this General‐to‐Clinical approach with a baseline strategy that involves pre‐training the model solely on ImageNet‐22K. To enable a fair comparison, we also pretrain a Swin‐L backbone using UM‐MAE on ImageNet‐22K as a comparison for pretraining strategies. For architectural comparison, we incorporate the well‐established ResNet18 [39] model, which we pretrain on ImageNet‐22K.

### 4.8 Genotyping and Construction of Polygenic Risk Scores

Peripheral blood samples from AZ‐GEV participants were genotyped using the Infinium Asian Screening Array (ASA) by Illumina. Standard quality control was applied using PLINK2 [52], including filters for only keeping those variants with a variant call rate (≥ 0.95), Hardy–Weinberg equilibrium (P ≥ 1×10^−6^), and minor allele frequency (MAF ≥ 0.01). Genotype imputation was performed on the Michigan Imputation Server [53] with the Haplotype Reference Consortium panel (HRC r1.1 2016), using Eagle v2.4 for phasing and Minimac4 for imputation. Variants with imputation INFO ≤0.8 were excluded, leaving 5,016,187 high‐quality variants for polygenic risk score (PRS) calculation.

PRS were constructed for each individual using summary statistics from previously published 679 PRSs relevant to 26 biomarker traits from PGS Catalog. These PRSs were originally developed across diverse ancestral populations and analytical settings, allowing us to benchmark face‐based prediction against a broad spectrum of existing genetic prediction models rather than a single ancestry‐restricted PRS. For each PRS, scores were calculated as the weighted sum of risk alleles carried by each individual, with weights downloaded from PGS Catalog, and the PRS scores were calculated using PLINK2. For each biomarker, we first evaluated the predictive performance of all available multi‐ancestry PRSs and identified the best‐performing PRS as the representative genetic prediction model. This best‐performing PRS was then compared with the MedicalFaceFound‐based prediction for the corresponding biomarker.

### 4.9 Development of Risk Evaluation Models

We constructed three stenosis‐classification models to evaluate the risk evaluation utility of MedicalFaceFound‐estimated biomarker values compared with conventional laboratory measurements.

Model 1 incorporated demographic and anthropometric variables (age, sex, height, weight) with laboratory‐measured lipid profiles (LDL‐C, HDL‐C, TC, TG). Model 2 replaced laboratory‐measured lipids with their face‐estimated counterparts. Model 3 expanded the feature set to include 16 face‐estimated cardiovascular biomarkers, thereby evaluating their incremental contribution in the absence of corresponding laboratory measurements in the DX‐EV cohort (Figure 4c).

All models were trained on the AZ‐TR cohort and externally validated in the DX‐EV cohort using a logistic model, with stenosis severity (≥ 50% vs. < 50%) as the clinical endpoint. Multivariable logistic regression was used for model development. To quantify the contribution of individual biomarkers, we used relative importance analysis to compare the contribution of face‐estimated and laboratory‐measured biomarkers to stenosis prediction with the “relaimpo” package [54]. Briefly, we fitted separate regression models for coronary stenosis using MedicalFaceFound‐estimated biomarker values and laboratory‐measured biomarkers as predictors, respectively. For each model, the total variance explained was decomposed into predictor‐specific components using the LMG method. The contribution estimates were then normalized to sum to 1 within each model, representing the relative share of explained variance attributable to each biomarker.

### 4.10 Test‐Retest Analysis

To assess the stability and reproducibility of our facial foundation model, we conducted a test–retest analysis across both internal and external cohorts. For each individual with multiple facial photographs collected, we randomly sampled two images taken on different occasions to form a test–retest pair. For each biomarker, the MedicalFaceFound model and other baseline models were applied independently to both images within each pair, and repeated predictions were obtained. Test–retest reliability was assessed using the intraclass correlation coefficient (ICC) for the repeated estimated biomarker values from each prediction model. ICCs were calculated with 95% confidence intervals using the “pingouin” package (v0.5.5).

### 4.11 Label Efficiency

Label efficiency pertains to the quantity of training data and annotations needed to attain a specified performance level for supervised fine‐tuning in a particular downstream task, reflecting the annotation effort required from medical professionals [55]. To assess the label efficiency of MedicalFaceFound relative to baseline architectures, we systematically varied the amount of supervised fine‐tuning samples in the AZ‐TR cohort. Training subsets were randomly down‐sampled to 400, 800, and 1600 samples without replacement, while the validation and internal testing cohorts were used in the same manner as in the former supervised fine‐tuning. Six representative blood biomarkers were selected to cover diverse physiological systems, and models were fine‐tuned following the predefined supervised fine‐tuning strategy.

In total, 558 models were trained, including 186 MedicalFaceFound models (62 biomarkers × 3 data regimes) and 372 baseline models (Swin‐Large and ResNet18, each trained under the same conditions). Model performance was evaluated on both the internal and three independent external cohorts (AZ‐EV1, AZ‐EV2, and DX‐EV). This design enabled a direct comparison of predictive performance under varying data regimes, thereby quantifying the label efficiency of MedicalFaceFound vs. conventional models.

### 4.12 Explanations for Fine‐Tuned Models

We applied Gradient‐weighted Class Activation Mapping (Grad‐CAM) to visualize the predictive mechanisms of fine‐tuned models for biomarker estimation. Grad‐CAM is a gradient‐based interpretability method that generates class‐discriminative localization maps by weighting activations at a selected layer with the gradients of a target output, thereby highlighting input regions most influential for prediction. For each fine‐tuned model, the first LayerNorm layer in the final block of each stage (i.e., the 2nd, 4th, 22nd, and 24th blocks) was designated as the target layer, and the single continuous output was treated as the target “class.”

### 4.13 Mobile Application Deployment and Validation

We developed a WeChat‐based mobile application using native WeChat components and the Vant Weapp framework to support user interaction and facial image collection. The backend was implemented in Golang with MySQL and Redis for data management and caching. MedicalFaceFound was deployed on an Ubuntu 22.04 server with an NVIDIA GeForce RTX 4090 GPU, where the backend was compiled into a standalone binary and managed via Systemd. Secure communication was ensured through HTTPS encryption, JWT‐based authentication, and SSL‐encrypted database connections. Further details are provided in the Resource Efficiency section of the Supplementary Information, with computational complexity and resource‐efficiency metrics summarized in Table S22.

To conduct a proof‐of‐concept evaluation of the use of smartphone‐acquired facial photographs, we recruited an independent study cohort. Facial images were collected by a physician who was blinded to the study design (N = 110), using the front‐facing camera of an iPhone 15 Pro Max. Other than a plain white background to ensure basic consistency, no restrictions were imposed on the shooting environment. One frontal image was collected per participant, without constraints on facial expression. This cohort was used to evaluate model performance on smartphone‐acquired images under physician‐assisted conditions. Given the small, single‐center cohort and the use of server‐side inference, this analysis was intended as a preliminary technical feasibility assessment rather than a comprehensive validation of mobile deployment.

### 4.14 Statistical Analysis

Baseline characteristics were summarized as mean (standard deviation, SD) for normally distributed continuous variables, median (interquartile range, IQR) for non‐normally distributed continuous variables, and frequency (percentage) for categorical variables. For each biomarker, predictive performance was evaluated by calculating Pearson's correlation coefficient between the estimated and measured biomarker values. Confidence intervals for Pearson's *r* were estimated using Fisher's *z*‐transformation. P values were adjusted for multiple testing using the Benjamini‐Hochberg false discovery rate (FDR‐BH) method. In addition to Pearson's *r*, continuous prediction performance was assessed using MAE, MSE, NRMSE (RMSE divided by the cohort‐specific mean of the observed biomarker value), and R^2^. Biomarkers with adjusted P ≥ 0.05 were considered to have no significant predictive ability. We conducted three complementary analyses to assess whether MedicalFaceFound captured biomarker‐related information beyond age, sex, height, weight, and BMI. First, for each of the 58 non‐demographic biomarkers, we compared a demographic‐only linear regression model with an extended model that additionally included the corresponding model‐derived biomarker value. Incremental performance was quantified as the increase in variance explained (R^2^), and the statistical significance of the two nested models was compared using a partial F‐test. Second, the measured and model‐derived values of each biomarker were separately adjusted for age, sex, height, weight, and BMI using linear regression. Pearson's correlation coefficient was then calculated between the resulting residuals. Third, for eight representative biomarkers, correlations between measured and model‐derived values were evaluated within subgroups defined by age (<55, 55–70, and >70 years), sex, BMI category (<24, 24.0–27.9, and ≥28.0 kg/m^2^), and coronary stenosis status. Multiple testing was corrected using the Benjamini‐Hochberg false discovery rate (FDR‐BH) method. All tests were two‐sided, and an FDR‐adjusted P‐value < 0.05 was considered statistically significant. For association analysis between estimated facial values and laboratory measurements for stenosis, we used logistic regression with covariates for age, sex, height, weight, and BMI. A two‐sided P < 0.05 indicates statistical significance. For risk evaluation models, we assessed performance using the area under the curve (AUC) and the area under the precision‐recall curve (APR), as well as accuracy, sensitivity, and specificity, with optimal cut‐off values determined by the maximum Youden index. 95% CI for performance metrics was estimated using bootstrap resampling with 1,000 iterations. All statistical analyses were conducted in “Python” (v3.10.14) and “statsmodels” (v0.14.2). Figures were generated with the “matplotlib” package (v3.10.0).

## Author Contributions

T.X. and M.W. conceptualized the study and led the study design and analyses. L.L., Y.D., G.Z., Y.Z., S.L., J.W., and W.Z. collected and curated the data. T.X. and H.X. conducted the model training and model evaluation. T.X. analyzed the results and generated the figures. L.L., Y.D., Y.Z. applied clinical deployment of the model and collected the data. X.C. and S.L. curated PRS. T.X., H.X., F.W., and M.W. co‐wrote the manuscript. H.Z., J.H., Y.Z., F.W., S.S., and H.H. critically revised the manuscript for important intellectual content. H.Z. and H.H. performed the technical validation and review. H.H., M.W., and Y.Z. jointly supervised the study. All authors discussed the results and provided comments on the manuscript.

## Code Availability

All software used in this study is publicly available. The custom code developed and used to train and fine‐tune the MedicalFaceFound model for biomarker prediction, as well as the analysis pipelines used in this paper, are available at https://github.com/1511878618/MedicalFaceFound.

## Conflicts of Interest

The authors declare no conflicts of interest.

## Supporting information




**Supporting File 1**: advs77861‐sup‐0001‐SuppMat.docx.

**Supporting File 2**: advs77861‐sup‐0002‐SuppMat.xlsx.

## Data Availability

All clinical data and facial images from the hospital‐based cohorts were collected and used with the informed consent of all participants. The study was conducted in accordance with the principles of the Declaration of Helsinki and was approved by the Institutional Review Board of Anzhen Hospital (Ethical Approval No. 2022211X). Access to raw facial images is restricted to protect participant privacy and can be granted only to qualified investigators with appropriate institutional and ethical approvals and a justified scientific purpose. The estimated biomarker scores, polygenic risk scores (PRS), and corresponding labels that do not contain any personally identifiable information are available upon reasonable request to the corresponding author. The ImageNet‐22K dataset used for initial pretraining was obtained from the official ImageNet repository (https://www.image‐net.org/), and the VGGFace2‐HQ dataset used for subsequent face‐domain pretraining was obtained from the VGGFace2‐HQ repository (https://github.com/NNNNAI/VGGFace2‐HQ). The PGS variant‐level weights used in this study are available for download through the PGS Catalog (https://www.pgscatalog.org/).
