# Supplementary material for: A Facial Foundation Model for Clinical Biomarker Prediction and Real‐World Mobile Deployment
Source: Adv Sci (Weinh). 2026 Oct 1:e77861. Online ahead of print. doi: 10.1002/advs.77861 (PMC13631392; doi:10.1002/advs.77861)
Supplement: Supplementary file 1 [file ADVS-9999-e77861-s002.docx]

# Supplementary Figures

**Supplementary Fig. 1 | General-to-clinical pre-training of MedicalFaceFound for biomarker prediction. a,** Schematic overview of the general-to-clinical pre-training strategy. The model was developed using a hierarchical general-to-clinical pre-training strategy. Stage 1: initialization with large-scale natural image data (14,197,122 photos, ImageNet-22K); Stage 2: adaptation to high-quality facial image datasets (1,160,250 photos, VGGFace2-HQ); and Stage 3: refinement with clinical facial cohorts (88,489 facial images on AZ-TR). Pre-training employed an encoder–decoder masked autoencoding scheme to learn transferable facial representations. After pre-training, the model was transferred for biomarker prediction (bottom). **b,** Reconstruction performance of models trained with different pre-training strategies. Reconstruction performance is evaluated using mean absolute error (MAE), with lower values indicating better reconstruction. MedicalFaceFound achieves the lowest reconstruction error compared with models pre-trained on ImageNet alone or further adapted using VGGFace2-HQ or AZ clinical facial images. **c,** Transfer of the pre-trained facial foundation model to downstream clinical prediction tasks. Facial images are encoded by the foundation model, and task-specific prediction heads are trained to estimate 62 biomarkers across eight physiological domains, including anthropometrics, hematology, cardiovascular, renal, endocrine, digestive, immune, and electrolyte systems.

**Supplementary Fig. 2 | Overview of data collection and preprocessing.** The figure summarizes the timeline and sources of data collection across multiple hospitals, including Anzhen Hospital (training cohort AZ-TR and external validation cohorts AZ-EV1, AZ-EV2, and AZ-GEV), Anzhen Tongzhou Hospital (AZTZ-EV), and Daxing Hospital (DX-EV). Data preprocessing included patient ID extraction using optical character recognition (OCR), database matching, and manual correction, followed by standardized image processing steps: facial region detection, data alignment, removal of background noise and blur, retention of clear frontal face images, and cropping to 256 × 256 pixels.

**Supplementary Fig. 3 | Data flow and evaluation framework of MedicalFaceFound. a,** Participant-level partitioning of AZ-TR and construction of the clinical pre-training, validation, and internal test sets. Validation and test participants were excluded from clinical pre-training. **b,** Three-stage progressive pre-training using ImageNet-22K, VGGFace2-HQ and clinical facial images, followed by biomarker-specific fine-tuning and internal testing. **c,** Independent evaluation in six external cohorts that were not used during pre-training, fine-tuning, or model selection. N denotes the number of participants.

**Supplementary Fig. 4 | Performance of additional clinical indicators across internal and external cohorts.** **a–h,** Model-estimation performance for age (a), height (b), BMI (c), weight (d), Hcy (e), UA (f), Hb (g), and HbA1C (h). For each indicator, the left panel shows measured values plotted against MedicalFaceFound-derived values, with the fitted trend line indicating the direction of association. The right panel shows cohort-specific Pearson’s *r* estimates with 95% CIs. Points indicate Pearson’s *r*, and horizontal error bars indicate 95% CI; red diamonds indicate the pooled estimate across cohorts. Reported q values denote the significance of the pooled association after multiple-testing correction. Abbreviations: BMI, body mass index; Hcy, homocysteine; UA, uric acid; Hb, haemoglobin; HbA1C, glycated haemoglobin; CI, confidence interval.

**Supplementary Fig. 5 | MedicalFaceFound improves biomarker prediction across cohorts and physiological systems. a,** Predictive performance of MedicalFaceFound, Swin-Large, and ResNet18 across eight physiological systems, including anthropometrics, renal, hematology, cardiovascular, immune, digestive, endocrine, and electrolyte. Performance is shown separately for the internal AZ-TR cohort and for the external cohorts, including AZ-EV1, AZ-EV2, AZTZ-EV, and DX-EV. Bars denote the mean predictive power within each physiological system, measured as Pearson’s *r* between estimated and measured values; error bars indicate variability across traits within each physiological system. **b,** Number of biomarker prediction tasks for which each model achieved the best-performing result. For each biomarker task, models were compared within each cohort and physiological system using Pearson’s *r*, and the model with the highest predictive performance was counted across cohorts and biomarkers.

**Supplementary Fig. 6 | Contribution of face components and demographic factors to biomarker prediction. a,** Stacked bar plots showing the variance explained by facial features, age, sex, height, weight, and BMI to the prediction of 26 biomarkers that were independently associated with laboratory measurements after adjustment by age, sex, height, weight, and BMI, evaluated in the AZ-EV1 cohort (N = 1,498). Each bar represents one biomarker, and the stacked colors denote the percentage contribution of each factor. **b,** Variance explained across all 26 biomarkers. Each dot represents a single biomarker, and the horizontal bars denote the mean. Relative importance was calculated as the variance explained by each focal predictor compared with the total variance explained by all predictors in the model.

**Supplementary Fig. 7 | Incremental predictive value of face-predicted biomarkers beyond demographic variables.** The heatmap shows the incremental R^2^ contributed by MedicalFaceFound-predicted biomarker values beyond age, sex, height, weight, and BMI in AZ-TR, AZ-EV1, and AZ-EV2. Cell values and color intensity indicate the magnitude of the incremental R^2^. Statistical significance was assessed after joint FDR correction across biomarkers and cohorts. Biomarker names shown in red indicate the eight representative biomarkers highlighted in the main manuscript. *, FDR < 0.05; **, FDR < 0.01; ***, FDR < 0.001. BMI, body mass index; FDR, false discovery rate; R^2^, coefficient of determination.

**Supplementary Fig. 8 | Residual correlations after adjustment for demographic variables.** Heatmaps show the raw (left) and residual (right) correlations between MedicalFaceFound-predicted and laboratory-measured biomarker values for AZ-TR, AZ-EV1, and AZ-EV2. Residuals were obtained after independently adjusting predicted and measured values for age, sex, height, weight, and BMI. Cell values represent Pearson’s *r*, and color intensity indicates the magnitude of the correlation. Statistical significance was assessed after joint FDR correction across biomarkers and cohorts. Biomarker names shown in red indicate the eight representative biomarkers highlighted in the main manuscript. *, FDR < 0.05; **, FDR < 0.01; ***, FDR < 0.001. BMI, body mass index; FDR, false discovery rate.

**Supplementary Fig. 9 | Comparison of raw and residual correlations after adjustment for demographic variables.** Scatter plots compare raw Pearson correlations with residual correlations between MedicalFaceFound-estimated and laboratory-measured biomarker values in AZ-TR, AZ-EV1, and AZ-EV2. Residuals were obtained after independently adjusting model-estimated and measured values for age, sex, height, weight, and BMI. Red points indicate residual correlations that remained significant after FDR correction (FDR < 0.05), whereas grey points indicate non-significant correlations. Error bars indicate 95% confidence intervals, and the dashed diagonal line denotes equality between raw and residual correlations. BMI, body mass index; FDR, false discovery rate.

**Supplementary Fig. 10 | Predictive information in MedicalFaceFound-estimated biomarker values beyond demographic variables.** Summary of the primary, residual-correlation, and incremental R^2^ analyses for 44 biomarkers across AZ-TR, AZ-EV1, and AZ-EV2. Raw r represents the correlation between face-estimated and measured values. Residual r represents their correlation after both values were independently adjusted for age, sex, height, weight, and BMI. Incremental R^2^ represents the additional variance explained by face-estimated values beyond these demographic variables. Pale-red cells indicate FDR-adjusted P < 0.05, white cells indicate non-significant results, and hatched cells indicate unavailable estimates. Red dots denote 20 biomarkers supported by three analyses in all three cohorts. Yellow dots denote 15 additional biomarkers supported by three analyses in at least one cohort. BMI, body mass index; FDR, false discovery rate; r, Pearson’s *r*; R^2^, coefficient of determination.

**Supplementary Fig. 11 | Subgroup analysis of MedicalFaceFound-estimated biomarker values.** Pearson correlations between MedicalFaceFound-estimated and measured Hb, HDL-C, HbA1C, and HCT values, stratified by age, sex, BMI, and stenosis status. Bars indicate Pearson correlation coefficients, and error bars indicate 95% confidence intervals. BMI, body mass index; Hb, haemoglobin; HDL-C, high-density lipoprotein cholesterol; HbA1C, glycated haemoglobin; HCT, haematocrit.

**Supplementary Fig. 12 | Pearson’s *r* of 679 PRS for 26 biomarkers in AZ-GEV cohort.** Boxplots showing the distribution of Pearson’s *r* for 679 polygenic risk scores (PRS) derived from the PGS Catalog from 26 biomarkers, tested in the AZ-GEV cohort (N = 111). Pearson’s *r* was calculated between measured biomarker values and PRS. Each box represents the interquartile range (IQR), with the horizontal line indicating the median, Pearson’s *r*, and whiskers denoting 1.5 × IQR.

**Supplementary Fig. 13 | Associations and distributions of cardiovascular-related biomarkers with coronary stenosis severity. a, b,** Forest plots showing odds ratios per SD (OR/SD) and 95% confidence intervals for 16 cardiovascular-related biomarkers in relation to coronary stenosis severity (<50% vs ≥50%) in the AZ-TR and DX-EV cohorts. Top, OR/SDs derived from laboratory measurements; bottom, OR/SD derived from facially estimated biomarker values. Each point represents the estimated OR/SD, with horizontal lines denoting 95% confidence intervals; the vertical dashed line indicates the null (OR/SD = 1). **c,** Violin plots showing normalized values of 16 cardiovascular-related biomarkers stratified by facial predictions and laboratory measurements across stenosis groups (<50% vs ≥50%). The width of each violin reflects data density; central black bars indicate interquartile ranges and white dots denote medians. Biomarkers include BNP, CK, CK-MB, D-dimer, HDL-C, HbA1C, Hcy, LDH, LDL-C, Lp(a), TC, TG, hs-CRP, hs-TnI, non-HDL, and sdLDL. Asterisks indicate the statistical significance of group comparisons: ns, *P* ≥ 0.05; *, *P* < 0.05; **, *P* < 0.01; ***, *P* < 0.001. Abbreviations: sdLDL, small dense low-density lipoprotein; non-HDL, non-high-density lipoprotein cholesterol; hs-TnI, high-sensitivity troponin I; hs-CRP, high-sensitivity C-reactive protein; TG, triglycerides; TC, total cholesterol; Lp(a), lipoprotein(a); LDL-C, low-density lipoprotein cholesterol; LDH, lactate dehydrogenase; Hcy, homocysteine; HbA1C, glycated hemoglobin A1c; HDL-C, high-density lipoprotein cholesterol; D-Dimer, D-dimer; CK-MB, creatine kinase-MB isoenzyme; CK, creatine kinase; BNP, B-type natriuretic peptide.

**Supplementary Fig. 14 | Label efficiency of MedicalFaceFound in biomarker prediction.** We evaluated label efficiency by varying the fraction of labeled training data used for model development and measuring the performance achieved at each level. For six exemplary biomarkers (eGFR (CKD-EPI), HbA1C, Hb, LYM, BMI, and HDL-C), MedicalFaceFound consistently outperformed Swin-Large and ResNet18, particularly when only small amounts of data were available. The dashed black lines mark the percentage of data required for MedicalFaceFound to match the performance of the Swin-Large or ResNet18 baseline model—10% for eGFR (CKD-EPI) and LYM, 20% for HbA1C, 29% for BMI, 63% for Hb, and 55% for HDL-C, respectively. Collectively, these results highlight the ability of MedicalFaceFound to achieve benchmark or superior performance with substantially fewer labeled samples, compared with the other two models. Points and lines represent the median Pearson’s *r* across four external cohorts (AZ-EV1, AZ-EV2, AZTZ-EV, and DX-EV).

**Supplementary Fig. 15 | Qualitative results of MedicalFaceFound. a,** Reconstructed facial images from highly masked images in a pretext task. Even with limited visible patches, MedicalFaceFound successfully recovered salient facial abnormalities, such as periorbital swelling, dental malocclusion, and macular lesions, indicating that the model has learned to capture disease-associated facial patterns. Note: these facial images were not included in our MedicalFaceFound pre-training process. **b,** Stage-specific representations of MedicalFaceFound for biomarker estimation. Grad-CAM heatmaps highlight the regions contributing to model decisions at different stages of the Swin-based architecture. Distinct patterns of feature utilization were observed across biomarkers: for eGFR prediction, early stages captured global facial context, intermediate stages emphasized the eyes and cheeks, and the final stage focused on the periocular region; for Hb prediction, later stages integrated signals from the entire face; for HDL-C, salient features were largely distributed around the eyes. Grad-CAM heatmaps indicate image regions contributing to model outputs and should not be interpreted as evidence of causal relationships or biological mechanisms. Abbreviations: eGFR, estimated glomerular filtration rate; Hb, hemoglobin; HDL-C, high-density lipoprotein cholesterol. Representative facial images shown in the figure were obtained from publicly available internet sources and are used for illustrative purposes only.

**Supplementary Fig. 16 | Overview of the general-to-clinical pre-training of MedicalFaceFound.** Images are sampled from ImageNet-22K, VGGFace2-HQ, and AZ-TR-unfiltered based on the probabilities indicated by pink squares, blue circles, and orange triangles, respectively. Following data augmentation, each image is masked at a 75% patch ratio and encoded by Swin-L into latent representations of dimension $\left[ B,8\times8,1536 \right]$, from which a lightweight decoder subsequently reconstructs the images. The reconstruction loss $L_{MAE}$ aligns the reconstructed images $\hat{x}$ with locally normalized targets $T\left( x \right)$, and the L2-SP loss $L_{L2-SP}$ restricts parameter drift from the reference $\theta^{0}$. Both the encoder and decoder are jointly optimized using $L=L_{MAE}+\alpha L_{L2-SP}$.

**Supplementary Fig. 17 | Overview of the fine-tuning and prediction workflow.** A facial image is processed by the Swin-L encoder (initialized from general-to-clinical pre-training) to extract feature representations of dimension $\left[ B,8\times8,1536 \right]$. A dedicated task head $H$ then maps these representations to a scalar prediction for a specific clinical biomarker. To prevent interference between different physiological targets, we independently fine-tune a complete model (Swin-L encoder and task head) for each of the $K$ biomarkers. Consequently, predicting the full biomarker panel entails $K$ parallel forward passes.

# Supplementary Materials

## Model Architecture

The MedicalFaceFound model utilizes a Swin-Large (Swin-L) Transformer as its core backbone to extract hierarchical facial representations. As illustrated in Supplementary Fig. 16, this encoder is structured into four hierarchical levels with varying numbers of Transformer blocks to capture visual features across different scales. During the general-to-clinical pre-training, the architecture integrates a lightweight reconstruction decoder following the UM-MAE framework. This simple decoder receives the latent features generated by the encoder and maps them back into the pixel space to complete the image reconstruction process. For downstream fine-tuning, MedicalFaceFound discards the pre-training decoder and appends a dedicated task head to the encoder to predict each clinical biomarker. As shown in Supplementary Fig. 17, this task head consists of a global average pooling layer, a layer normalization module, and a multilayer perceptron. We independently fine-tune the entire encoder and its corresponding task head for each biomarker to construct a dedicated prediction model.

## General-to-Clinical Pre-training

As illustrated in Supplementary Fig. 1 and Supplementary Fig. 3, MedicalFaceFound employs UM-MAE to progressively acquire general visual priors, facial morphology, and clinical facial phenotypes across three successive stages. Let $\mathcal{D}_{G}$, $\mathcal{D}_{F}$, and $\mathcal{D}_{C}$ denote the ImageNet-22K (14,197,122 images), VGGFace2-HQ (1,160,250 images), and AZ-TR-unfiltered (88,489 images) datasets, respectively. We define the trainable parameters as $\theta=\left\{ \theta_{\mathrm{enc}},\theta_{\mathrm{dec}} \right\}$, corresponding to the encoder and decoder. During stage transitions, the current parameters $\theta$ and their reference anchors $\theta^{0}$ are initialized to $\mathbf{0}$ for Stage 1 and to the final checkpoint $\theta^{*}$ of the preceding stage for Stages 2 and 3.

To prevent catastrophic forgetting and stabilize representation transfer during domain adaptation, we implement a historical-sample replay strategy. This approach maintains continuous exposure to earlier domains by mixing datasets during batch generation. Ordering the datasets as $\left\{ \mathcal{D}_{G},\mathcal{D}_{F},\mathcal{D}_{C} \right\}$, the stage-specific sampling probabilities are defined as:

$\boldsymbol{\pi}^{\left( 1 \right)}=\left\{ 100\%,0,0 \right\},\quad\boldsymbol{\pi}^{\left( 2 \right)}=\left\{ 10\%,90\%,0 \right\},\quad\boldsymbol{\pi}^{\left( 3 \right)}=\left\{ 10\%,10\%,80\% \right\}.$

For a given stage, we designate the dataset with the highest sampling probability as the primary dataset $\mathcal{D}$. A training batch $\mathcal{B}$ is formulated by randomly drawing images without replacement from the available datasets based on $\boldsymbol{\pi}^{\left( r \right)}$:

$\mathcal{B}\sim\mathrm{sample} \left( \boldsymbol{\pi}^{\left( r \right)} \right).$	(1)

This batch generation process continues for $E_{r}$ epochs per stage, with a single epoch concluding only after all images in the primary dataset $\mathcal{D}$ have been sampled exactly once.

To enhance model robustness against acquisition variability while preserving essential facial structures and clinically relevant semantics, we apply standard random image augmentations in Stage 1 alongside mild transformations including random-resized cropping, horizontal flipping, and weak color jitter in Stages 2 and 3. For a raw image $\tilde{x}\in\mathcal{B}$ and an augmentation operator $A$ drawn from a predefined augmentation family $\mathcal{A}$, the augmented input is formulated as:

$A\sim\mathcal{A},\quad\quad x=A\left( \tilde{x} \right).$	(2)

Furthermore, we introduce local target normalization to mitigate the dominance of global illumination, skin tone, and background colors. The normalized target is computed as:

$T\left( x \right)=\frac{x-\mu_{\mathrm{loc}}\left( x \right)}{\sigma_{\mathrm{loc}}\left( x \right)+\epsilon},$	(3)

where $\mu_{\mathrm{loc}}\left( x \right)$ and $\sigma_{\mathrm{loc}}\left( x \right)$ represent the channel-wise local mean and standard deviation obtained via a sliding window, and $\epsilon$ ensures numerical stability. This normalization is strictly applied to the reconstruction target, leaving the encoder input untouched and compelling the model to focus on localized facial features.

During the forward pass, we generate a random block-wise mask $m$ to obscure 75% of the patches in the augmented image $x$. The encoder processes the visible regions, and the decoder reconstructs the full image $\hat{x}$:

$\hat{x}=D_{\theta_{\mathrm{dec}}}\left( E_{\theta_{\mathrm{enc}}}\left( \mathrm{mask} \left( x \right) \right) \right).$	(4)

The self-supervised reconstruction objective $\mathcal{L}_{\mathrm{MAE}}$ penalizes the absolute differences between the reconstructed output and the locally normalized target exclusively within the masked regions:

$\mathcal{L}_{\mathrm{MAE}}=\mathrm{mean} \left( m\odot\left| T\left( x \right)-\hat{x} \right| \right),$	(5)

where $\odot$ denotes element-wise multiplication. To further constrain parameter drift when transitioning between domains, we incorporate L2-SP regularization:

$\mathcal{L}_{L2-SP}=\left\| \theta-\theta^{0} \right\|_{2.}$	(6)

By establishing $\theta^{0}=0$ in Stage 1, this term functions as standard L2 weight decay. In Stages 2 and 3, $\theta^{0}$ acts as a soft anchor based on the previous stage's checkpoint. Finally, we optimize the complete parameter set $\theta$ using AdamW with layer-wise learning-rate scaling. The joint objective and update rule are expressed as:

$\mathcal{L}=\mathcal{L}_{\mathrm{MAE}}+\alpha\mathcal{L}_{L2-SP},\quad\quad\theta_{\mathcal{l}}\leftarrow\theta_{\mathcal{l}}-\eta\gamma_{\mathcal{l}}\nabla_{\theta_{\mathcal{l}}}\mathcal{L},$	(7)

where $\alpha$ is a balancing coefficient, $\eta$ is the base learning rate, and $\gamma_{\mathcal{l}}$ modulates the learning rate for layer $\mathcal{l}$.

Integrating the progressive data sampling, dynamic masking, and the regularized optimization objective, we summarize the complete general-to-clinical pre-training workflow in Algorithm 1.

**Algorithm 1 | General-to-Clinical Pre-training of MedicalFaceFound**

**Input:** datasets $\left\{ \mathcal{D}_{G},\mathcal{D}_{F},\mathcal{D}_{C} \right\}$, sampling probabilities $\left\{ \boldsymbol{\pi}^{\left( 1 \right)},\boldsymbol{\pi}^{\left( 2 \right)},\boldsymbol{\pi}^{\left( 3 \right)} \right\}$, and stage epoch counts $\left\{ E_{1},E_{2},E_{3} \right\}$

**Output:** final pre-trained parameters $\theta^{*}$

1.	**for** $r=1$ **to** $3$ **do**

2.	initialize $\theta$ and $\theta^{0}$ for the current stage

3.	set $\mathcal{D}$ as the primary dataset

4.	**for** $e=1$ **to** $E_{r}$ **do**

5.	**while** $\mathcal{D}$ has not been fully sampled in the current epoch **do**

6.	sample a batch $\mathcal{B}$	// Eq. (1)

7.	obtain augmented images $\mathcal{X}=\left\{ x\mid x=A\left( \tilde{x} \right), \tilde{x}\in\mathcal{B} \right\}$	// Eq. (2)

8.	obtain $\mathcal{T}=\left\{ T\left( x \right)\mid x\in\mathcal{X} \right\}$ and $\hat{\mathcal{X}}=\left\{ \hat{x}\mid x\in\mathcal{X} \right\}$	// Eqs. (3) and (4)

9.	compute loss $\mathcal{L}$ and optimize $\theta$	// Eqs. (5), (6), and (7)

10.	**end while**

11.	**end for**

12.	store the optimized parameters $\theta^{*}$

13.	**end for**

14.	**return** $\theta^{*}$

## Fine-tuning and Evaluation

Supplementary Fig. 17 illustrates our biomarker-specific fine-tuning and prediction workflow. Let $K=62$ denote the total number of clinical biomarkers, $\mathcal{X}$ the facial image space, and $\mathcal{Y}\subseteq\mathbb{R}^{K}$ the continuous label space. Each image $x\in\mathcal{X}$ is associated with a ground-truth vector $\mathbf{y}=\left( y_{1},y_{2},\ldots,y_{K} \right)\in\mathcal{Y}$, where $y_{k}$ represents the laboratory-measured value for the $k$-th biomarker. We define the labeled source dataset for fine-tuning as $\mathcal{D}_{\mathrm{src}}\subset\mathcal{X}\times\mathcal{Y}$, and the target dataset for internal or external evaluation as $\mathcal{D}_{\mathrm{tgt}}\subset\mathcal{X}\times\mathcal{Y}$. Specifically, we randomly partition the AZ-TR cohort into training (70%), validation (20%), and internal testing (10%) subsets. The training subset constitutes $\mathcal{D}_{\mathrm{src}}$, the validation subset is utilized for checkpoint selection, and the internal test set alongside four independent external cohorts function as distinct $\mathcal{D}_{\mathrm{tgt}}$ configurations during evaluation.

General-to-clinical pre-training yields the optimized parameter set $\theta^{*}=\left\{ \theta_{\mathrm{enc}}^{*},\theta_{\mathrm{dec}}^{*} \right\}$. For downstream adaptation, we discard the decoder weights $\theta_{\mathrm{dec}}^{*}$ and retain the encoder weights $\theta_{\mathrm{enc}}^{*}$ as the initialization point. To prevent negative transfer across diverse physiological systems, we independently train $K$ complete models for the $K$ biomarkers, dedicating a separate Swin-Large encoder and task head to each. For a given biomarker $k\in\left\{ 1,\ldots,K \right\}$, the specific model parameters are denoted by $\theta_{k}=\left\{ \theta_{\mathrm{enc},k},\theta_{\mathrm{head},k} \right\}$, where $\theta_{\mathrm{enc},k}$ is initialized from $\theta_{\mathrm{enc}}^{*}$ and $\theta_{\mathrm{head},k}$ represents the randomly initialized task-specific prediction head $H$. The scalar prediction for biomarker $k$ is thus formulated as:

$\hat{y}_{k}=H_{\theta_{\mathrm{head},k}}\left( E_{\theta_{\mathrm{enc},k}}\left( x \right) \right).$	(8)

Given a training mini-batch $\mathcal{B}\sim\mathcal{D}_{\mathrm{src}}$, the model is optimized using the Mean Squared Error (MSE) loss:

$\mathcal{L}_{\mathrm{MSE}}^{\left( k \right)}=\frac{1}{\left| \mathcal{B} \right|}\sum_{\left( x,\mathbf{y} \right)\in\mathcal{B}} \left( \hat{y}_{k}-y_{k} \right)^{2.}$	(9)

We jointly optimize the full encoder and the task head using the AdamW optimizer. To preserve the pre-trained representations in shallower layers while allowing task-specific adaptation in deeper layers, we apply layer-wise learning rate decay. The parameter update rule for layer $\mathcal{l}$ is governed by:

$\theta_{k\mathcal{,l}}\leftarrow\theta_{k\mathcal{,l}}-\eta\gamma_{\mathcal{l}}\nabla_{\theta_{k\mathcal{,l}}}\left( \mathcal{L}_{\mathrm{MSE}}^{\left( k \right)}+\lambda\mathcal{R}\left( \theta_{k} \right) \right),$	(10)

where $\mathcal{R}\left( \theta_{k} \right)$ denotes the weight decay regularization, $\lambda$ is the regularization coefficient, $\eta$ is the base learning rate, and $\gamma_{\mathcal{l}}\in(0,1]$ is the decay scale for layer $\mathcal{l}$. We employ an early stopping mechanism, terminating the fine-tuning process if the validation loss fails to improve for $P$ consecutive epochs.

During evaluation, the optimal checkpoint $\theta_{k}^{*}$ for each biomarker is applied to the target dataset $\mathcal{D}_{\mathrm{tgt}}$. The predictive performance is quantified using Pearson's correlation coefficient ($r$) between the predicted estimates ${\hat{\mathbf{y}}}_{k}$ and the clinical measurements $\mathbf{y}_{k}$. This evaluation is systematically conducted across the AZ-TR internal test set and the four independent external cohorts. The complete biomarker-specific fine-tuning and evaluation protocols are summarized in Algorithm 2 and Algorithm 3, respectively.

**Algorithm 2 | Biomarker-specific Fine-tuning of MedicalFaceFound**

**Input:** training data $\mathcal{D}_{\mathrm{src}}$, validation data, initialized parameters $\theta_{\mathrm{enc}}^{*}$, number of biomarkers $K$, maximum fine-tuning epoch $E_{\mathrm{ft}}$, and early-stopping patience $P$

**Output:** biomarker-specific parameters $\left\{ \theta_{k}^{*} \right\}_{k=1}^{K}$

1.	**for** $k=1$ **to** $K$ **do**

2.	initialize $\theta_{\mathrm{enc},k}$ from $\theta_{\mathrm{enc}}^{*}$ and initialize $\theta_{\mathrm{head},k}$ randomly

3.	**for** $e=1$ **to** $E_{\mathrm{ft}}$ **do**

4.	**for** each batch $\mathcal{B}\sim\mathcal{D}_{\mathrm{src}}$ **do**

5.	obtain predictions ${\hat{\mathbf{y}}}_{k}=\left\{ \hat{y}_{k}\mid\left( x,\mathbf{y} \right)\in\mathcal{B} \right\}$	// Eq. (8)

6.	compute regularized loss and update $\theta_{k}$	// Eqs. (9) and (10)

7.	**end for**

8.	evaluate validation loss and record the best checkpoint $\theta_{k}^{*}$

9.	**if** validation loss has not decreased for $P$ consecutive epochs **then**

10.	**break**

11.	**end if**

12.	**end for**

13.	**end for**

14.	**return** $\left\{ \theta_{k}^{*} \right\}_{k=1}^{K}$

**Algorithm 3 | Evaluation of MedicalFaceFound**

**Input:** target data $\mathcal{D}_{\mathrm{tgt}}$ and biomarker-specific parameters $\left\{ \theta_{k}^{*} \right\}_{k=1}^{K}$

**Output:** biomarker-specific correlation metrics $\left\{ \mathcal{M}_{r}^{\left( k \right)} \right\}_{k=1}^{K}$

1.	**for** $k=1$ **to** $K$ **do**

2.	extract predictions ${\hat{\mathbf{y}}}_{k}$ and measurements $\mathbf{y}_{k}$ for all samples in $\mathcal{D}_{\mathrm{tgt}}$	// Eq. (8)

3.	compute $\mathcal{M}_{r}^{\left( k \right)}=\mathrm{PearsonCorrelation} \left( {\hat{\mathbf{y}}}_{k},\mathbf{y}_{k} \right)$

4.	store $\mathcal{M}_{r}^{\left( k \right)}$

5.	**end for**

6.	**return** $\left\{ \mathcal{M}_{r}^{\left( k \right)} \right\}_{k=1}^{K}$

## Experimental Settings

We conducted the general-to-clinical pre-training on a dedicated Ubuntu 20.04.6 LTS server equipped with 2 AMD EPYC 7513 32-core processors (64 physical cores and 128 logical threads) and 8 NVIDIA GeForce RTX 3090 GPUs (24 GB memory per device). Operating within a software environment of PyTorch 2.3.0, CUDA 12.1, and cuDNN 8.9.2, we completed the entire pre-training process in approximately 2,700 hours. For subsequent biomarker-specific fine-tuning and evaluation, we transitioned to a separate Debian GNU/Linux 13 server powered by 2 Intel Xeon E5-2699 v4 processors (44 physical cores and 88 logical threads) and a NVIDIA GeForce RTX 4090 GPU (24 GB memory). On this system, we maintained an identical deep learning software stack (PyTorch 2.3.0, CUDA 12.1, and cuDNN 8.9.2) supported by NVIDIA driver 550.144.03. We detail the key experimental settings and hyperparameters corresponding to both the general-to-clinical pre-training and downstream fine-tuning phases in Supplementary Table 21.

**Supplementary Table 21 | Key experimental settings for MedicalFaceFound.**

| **Symbol** | **Definition** | **Value** |
| --- | --- | --- |
| **General-to-clinical pre-training** | | |
| $E$ | Number of training epochs per stage | 200 epochs |
| $B$ | Global batch size | 256 |
| $\eta$ | Initial learning rate | $2\times{10}^{-4}$ |
| $W$ | Number of warm-up epochs | 20 epochs |
| $\lambda_{\mathrm{wd}}$ | AdamW weight decay | $0.01$ |
| $\left( \beta_{1},\beta_{2} \right)$ | AdamW beta parameters | $\left( 0.9,0.999 \right)$ |
| $\rho$ | Training mask ratio | $0.75$ |
| $p$ | Mask-block size | $16\times16$ pixels |
| $\boldsymbol{\pi}$ | Dataset sampling probabilities for $\left( \mathcal{D}_{G},\mathcal{D}_{F},\mathcal{D}_{C} \right)$ | (Stage 1) $\left( 1.00,0,0 \right)$ (Stage 2) $\left( 0.10,0.90,0 \right)$ (Stage 3) $\left( 0.10,0.10,0.80 \right)$ |
| $\alpha_{\mathrm{SP}}$ | L2-SP coefficient/balance factor | $0.01$ |
| $\boldsymbol{\gamma}$ | Learning-rate scales for the four Swin-L stages | (Stage 1) $\left( 1.0,1.0,1.0,1.0 \right)$ (Stage 2) $\left( 0.75,0.75,1.0,1.0 \right)$ (Stage 3) $\left( 0.75,0.75,1.0,1.0 \right)$ |
| $p_{A}$ | Selection probability for each random augmentation | Random-resized cropping: $1/3$ Horizontal flipping: $1/3$ Color jitter: $1/3$ |
| $s$ | Random seed | 42 |
| **Biomarker-specific fine-tuning** | | |
| $E_{\mathrm{ft}}$ | Maximum number of fine-tuning epochs | 50 epochs |
| $B_{\mathrm{ft}}$ | Fine-tuning batch size | 32 |
| $\eta_{\mathrm{ft}}$ | Initial learning rate | $5\times{10}^{-3}$ |
| $W_{\mathrm{ft}}$ | Number of warm-up epochs | 10 |
| $\lambda_{\mathrm{ft}}$ | AdamW weight decay | $0.01$ |
| $\left( \beta_{1,\mathrm{ft}},\beta_{2,\mathrm{ft}} \right)$ | AdamW beta parameters | $\left( 0.9,0.999 \right)$ |
| $\boldsymbol{\gamma}_{\mathrm{ft}}$ | Layer-wise learning-rate decay scales | $\left( 0.75,0.75,1.0,1.0 \right)$ |
| $P$ | Early-stopping patience | 16 epochs |

## Resource Efficiency

We evaluate the computational complexity and real-world deployment feasibility of our approach in Supplementary Table 22. Given its reliance on the Swin-Large backbone, the computational requirements of MedicalFaceFound closely mirror those of a standard Swin-Large model. Therefore, we report the metrics for MedicalFaceFound as the Swin-Large baseline and compare them directly with a conventional lightweight ResNet18 architecture. We conducted all efficiency evaluations on the hardware environment used for downstream fine-tuning, which features an Intel Xeon E5-2699 v4 processor and an NVIDIA GeForce RTX 4090 GPU. We calculated metrics including floating-point operations (FLOPs), model file size, peak GPU memory allocation, and single-image inference time under FP32 precision, reporting the average values across 10,000 images with a batch size of 1. To estimate overall image throughput, we processed 10,000 images with a batch size of 32 to provide a practical performance reference for standard batch processing.

To prevent negative transfer across distinct physiological systems, we train a separate model for each of the 62 biomarkers. Consequently, predicting the complete clinical panel requires running multiple independent models. We therefore report the aggregate resource consumption required to process the full panel during inference. We calculated the total GPU memory consumption by multiplying the peak GPU memory of a single model by 62. To measure the total inference time for all 62 predictions, we designed a sequential evaluation pipeline. This pipeline loads a single image into the GPU, sequentially loads the 62 sets of model parameters into host memory before transferring them to the GPU, and executes the forward pass for each biomarker. We repeated this procedure 100 times and report the average duration, providing a transparent assessment of the computational demands for deploying the complete MedicalFaceFound suite.

**Supplementary Table 22 | Computational complexity and resource efficiency comparison.**

| **Resource Metric** | **ResNet18** | **MedicalFaceFound** |
| --- | --- | --- |
| Parameter count (M) | 11.18 | 194.94 |
| FLOPs (G) | 4.84 | 91.23 |
| Model file size (MB) | 44.79 | 779.90 |
| Single-image GPU inference time (ms) | 1.44 | 8.89 |
| Single-image CPU inference time (ms) | 20.32 | 412.17 |
| Peak GPU memory usage (MB) | 84.99 | 887.78 |
| Throughput (images/s) | 4163 | 289 |
| Total models to load | 62 | 62 |
| Total GPU memory consumption (MB) | 5269 | 55042 |
| Total inference time (ms) | 82.86 | 517.28 |
